# Cellular Distribution of Secreted Phospholipase A2 in Lungs of IPF Patients and Its Inhibition in Bleomycin-Induced Pulmonary Fibrosis in Mice

**DOI:** 10.3390/cells12071044

**Published:** 2023-03-30

**Authors:** Ashish Jaiswal, Rakhshinda Rehman, Joytri Dutta, Sabita Singh, Archita Ray, Malathy Shridhar, Jaswant Jaisankar, Manas Bhatt, Dikshit Khandelwal, Bandya Sahoo, Arjun Ram, Ulaganathan Mabalirajan

**Affiliations:** 1 Molecular Pathobiology of Respiratory Diseases, Cell Biology and Physiology Division, Council of Scientific and Industrial Research (CSIR)—Indian Institute of Chemical Biology (IICB), Kolkata 700091, India; 2 Academy of Scientific and Innovative Research (AcSIR), Sector-19, Kamla Nehru Nagar, Ghaziabad 201002, India; 3 Molecular Pathobiology of Respiratory Diseases, CSIR—Institute of Genomics and Integrative Biology, Mall Road, Delhi 110007, India; 4 Division of Pulmonary and Critical Care Medicine, Brigham and Women’s Hospital, Boston, MA 02115, USA; 5 The World Community Service Centre, Thiruvanmiyur, Chennai 600041, India; 6 Kalinga Institute of Medical Sciences, KIIT University, Bhubaneswar 751024, India

**Keywords:** idiopathic pulmonary fibrosis, parabromophenacyl bromide, secretory phospholipase A2, bleomycin, Group-II secretory phospholipase A2

## Abstract

Idiopathic pulmonary fibrosis (IPF) is a chronic lung disease with a very poor prognosis as it has a 2.5 to 5 years mean survival after proper diagnosis. Even nintedanib and pirfenidone cannot halt the progression, though they slow the progression of IPF. Hence, there is a need to understand the novel pathophysiology. Phospholipase A2 (PLA2) could be the ideal candidate to study in IPF, as they have a role in both inflammation and fibrosis. In the present study, we have shown the expression profile of various secretory Phospholipase A2 (PLA2) isoforms by analyzing publicly available transcriptome data of single cells from the lungs of healthy individuals and IPF patients. Among 11 members of sPLA2, PLA2G2A is found to be increased in the fibroblasts and mesothelial cells while PLA2G5 is found to be increased in the fibroblasts of IPF patients. We identified a subset of fibroblasts expressing high PLA2G2A with moderate expression of PLA2G5 and which are specific to IPF only; we named it as PLA2G2A+ IPF fibroblast. Pathway analysis revealed that these PLA2G2A+ IPF fibroblast have upregulation of both inflammatory and fibrosis-related pathways like the TGF-β signaling pathway, IL-17 signaling, the arachidonic acid metabolism pathway and ECM-receptor interaction. In addition to this, we found elevated levels of sPLA2-IIA in plasma samples of IPF patients in our cohort. PLA2G3, PLA2G10 and PLA2G12B are found in to be increased in certain epithelial cells of IPF patients. Thus, these findings indicate that these five isoforms have a disease-dominant role along with innate immune roles as these isoforms are found predominantly in structural cells of IPF patients. Further, we have targeted sPLA2 in mice model of bleomycin-induced lung fibrosis by pBPB, a known sPLA2 inhibitor. pBPB treatment attenuated lung fibrosis induced by bleomycin along with a reduction in TGF-β and deposition of extracellular matrix in lung. Thus, these findings indicate that these sPLA2 isoforms especially PLA2G2A may serve as a therapeutic target in lung fibrosis.

## 1. Introduction

Idiopathic pulmonary fibrosis (IPF) is a gradually progressive lung deteriorating disease with a very poor prognosis, with mere 2.5 to 5 years mean survival. IPF mostly affects older individuals; however, various studies had found that IPF may affect young individuals. IPF was considered an inflammatory disease principally. In contrast, recent literature based on clinical and experimental evidences emphasized that it could be an epithelial-driven disease [1,2,3]. The development of IPF features could be due to the convergence of multiple factors like genetics, aging, and environmental factors and this convergence could activate the epithelium to initiate the disease by secreting various profibrotic mediators that cause migration and activations of fibroblasts. This also causes conversion of fibroblasts into myofibroblasts that secrete abundant extracellular matrix proteins to cause lung fibrosis. Epithelial apoptosis in response to lung injury plays a crucial role in the development of lung fibrosis. LPA-LPA1 signaling is one of the pathways that are known to promote epithelial cell death with a resistance to fibroblast cell death; to promote the development of pulmonary fibrosis after lung injury [4]. LPA (lysophosphatidic acid), a pro-fibrotic, inflammatory mediator and also a downstream metabolite of Phospholipase A2 (PLA2) signaling was found to be increased in both BAL fluid of IPF patients and bleomycin-treated mice. LPA-LPA1 signaling has been shown to be crucial for fibroblast recruitment and vascular leakage in a bleomycin-induced mice model of pulmonary fibrosis [4,5,6]. This indicates that PLA2 may play a critical role in lung fibrosis via LPA-LPA1 signaling.

Phospholipase A2 (PLA2), key enzymes involved in breakdown of membrane phospholipids to release free fatty acids like arachidonic acid, has been shown to be the cause of lung injury and inflammation [7]. They are present in multiple forms: secretory phospholipase A2 (sPLA2), cytosolic phospholipase A2 (cPLA2), Ca^2+^ independent (iPLA2) and lipoprotein associated phospholipase A2, based on biochemical characteristics [7,8]. The cPLA2 null mice had shown attenuation of pulmonary fibrosis induced by bleomycin with reduction in thromboxane and leukotrienes [9]. The cPLA2 has been shown to be not only crucial in lung fibrosis but also in fibrosis of other organs. For example, high-fat-diet-fed mice are resistant to developing liver fibrosis in cPLA2 alpha knockout mice [10]. Indeed, cPLA2 KO mice had not merely reduced the fibrosis, but also inflammation, indicating that PLA2 is involved in early stages of fibrosis, as fibrosis is the end product of inflammation. This also indicates that cPLA2 may also be involved in the generation of pro-inflammatory mediators. In addition to cPLA2, it has been demonstrated that sPLA2-IIA promotes the conversion of fibroblast into myofibroblasts via EGFR activation to cause cardiac fibrosis [11]. Compared to cPLA2, certain sPLA2 isoforms are not only found in blood, but are also responsible for controlling other cells and foreign elements like bacteria, thanks to substrate availability in the bacterial membrane [12,13,14,15,16,17]. Thus, sPLA2 is not only important in prognosis of the disease, but also could be a therapeutic target.

In spite of its diversity, each PLA2 is unique, owing to different cellular distribution and substrate. Studying the status of one PLA2 alone is not sufficient to understand the complete pathophysiology of IPF. In addition, it had been demonstrated that exogenous sPLA2 had been shown to activate the expression of cPLA2 in neutrophils through LTB4 (Leukotriene B4) that leads to cause phosphorylation of cPLA2 [15]. Similarly, sPLA2 had been shown to activate cPLA2 in astrocytoma cells [16] and keratinocytes [17]. These indicate that sPLA2 could be an important upstream regulator of cPLA2. So, it could be important to study the status and cellular distribution of various forms of sPLA2. In this study, for the first time, we have elucidated the expression profile of various sPLA2 by analyzing transcriptome of single cells from control healthy individuals and IPF patients from three datasets that are publicly available as described by Habermann et al. [18], Tsukui et al. [19] and Reyfman et al. [20]. Single-cell transcriptomic data analysis revealed an increase in expression of PLA2G2A (protein name is sPLA2-IIA) and PLA2G5 (protein name is sPLA2-V) in a subset of fibroblasts of IPF patients as compared to healthy individuals. These fibroblasts are present in IPF individuals only and highly expressing PLA2G2A, so we named them as PLA2G2A+ IPF fibroblast. TGF-β signaling pathway, IL-17 signaling, arachidonic acid metabolism pathway, ECM-receptor interaction and other fibrosis-related pathways are upregulated in these PLA2G2A+ IPF fibroblast.

We have earlier explored the anti-asthma potential of parabromophenacyl bromide (pBPB), a known sPLA2 inhibitor both in the acute and chronic model of asthma [21,22]. Therefore, we have also studied the effect of pBPB on bleomycin-induced lung fibrosis in mice. Inhibition of sPLA2 by pBPB treatment had shown the attenuation of certain features of pulmonary fibrosis along with reduction in TGF-β and deposition of extracellular matrix in lung. These findings might be important in developing effective therapeutic strategies for IPF.

## 2. Materials and Methods

### 2.1. Single-Cell RNA-Sequencing Data Acquisition

We have used three datasets for data mining of publicly available data present on NCBI gene expression omnibus: (A) GSE135893, (B) GSE132771 and (C) GSE122960 of single-cell RNA sequencing that was reported by Habermann et al. [18], Tsukui et al. [19] and Reyfman et al. [20], respectively. We acquired Single-Cell transcriptome data of eight, three, and six healthy individuals (control) and eight, three and four IPF patients (IPF) from datasets A–C, respectively (Supplementary Tables S1 and S2). As mentioned in the original publications, all the studies requiring the clinical sample were approved by Institutional Review Board. For diagnosis of pulmonary fibrosis, they had used American Thoracic Society/European Respiratory Society criteria [18,19,20]. The detailed description on acquiring the lung samples from healthy individuals and IPF patients were are available in the original publications. The demographic details of all clinical samples as described by Habermann et al. [18] and Reyfman et al. [20] can be found on Supplementary Tables S1 and S2, respectively.

### 2.2. Single-Cell RNA-Sequencing Data Analysis

All algorithm used for analysis of single cell RNA seq data was based on Seurat; R toolkit for single cell genomics with custom modification [23,24,25,26]. Seurat version 4 was used for analysis and visualization of single-cell RNA-sequencing data on R Studio. The combined raw data of all the samples obtained from the gene expression omnibus were read on RStudio and Seurat object for the same has been created. For dataset A, a subset for the datasets of eight controls and eight IPF patients with 73,778 cells were created and subject identifier information has been added to the metadata. However, individual cells with less than 200 or more than 4500 unique gene count and more than 25% of reads arising from mitochondrial genes were filtered out. Normalization and scaling were done using SCTransform that normalizes the single cell UMI-data by variance-stabilizing transformation [27]. The principal component analysis (PCA) was used for dimensional reduction based on the top 3000 most variable genes. Variable genes were determined using the *FindVariableGenes* function of Seurat. The *FindClusters* function of Seurat was used on PCA reduced genes (dims = 1:20). Uniform manifold approximation and projection (UMAP) was used for visualization of clusters on a 2-D map. Differentially expressed genes were determined by the *FindMarkers* function of Seurat, which is based on the non-parametric Wilcoxon Rank sum test. To further study the status of various sPLA2, a feature plot/dot plot/violin plot was generated for each subgroup of Seurat objects. The *FindMarkers* function of Seurat was used to determine all the differentially expressed genes on a subcluster of fibroblast (PLA2G2A IPF fibroblast), highly expressing PLA2G2A.

Two more datasets were analyzed for validation of dataset A. Dataset B and C were also analyzed using Seurat version 4 on RStudio. For analysis of dataset B, cells with less than 300 unique gene and more than 10% mitochondrial gene were excluded. For dataset C analysis, cells with less than 300 unique gene and more than 35% mitochondrial gene were excluded. Log-normalization was performed. FindVariableGenes function of Seurat was used to detect variable genes on dimensionality reduced data. On PCA-reduced genes, graph-based clustering was performed using the FindClusters function of Seurat (at resolution = 0.6 and dims = 1:20). UMAP was used for visualization of clusters on a 2-D map. Differentially expressed genes were determined by the *FindMarkers* function of Seurat, which is based on a non-parametric Wilcoxon Rank sum test. Mesenchymal cells were identified by canonical markers (Supplementary Figure S3A–D and Supplementary Figure S5A–D for data set B and C respectively) and further analyzed. Based on expression of markers, cells were annotated (Supplementary Figures S4 and S3A and Supplementary Figures S6 and S4A for data set B and C respectively). To further validate the results for dataset A, UMAP, feature plot and violin plot were created for PLA2G2A and violin plot for PLA2G5.

### 2.3. Pathway Enrichment Analysis

*FindMarkers* function of Seurat was used to determine all the differentially expressed genes on a subcluster of fibroblast (PLA2G2A IPF fibroblast), highly expressing PLA2G2A. All the significantly differentially expressed genes in PLA2G2A+ IPF fibroblast as compared to all other mesenchymal cells with logFC > 0.35 were used as an input for KEGG pathway enrichment analysis using the most widely used gene set enrichment tool WebGestalt 2019 [28]. Statistical significance of pathways was based on *p*-value. The normalized enrichment score and *p*-value of all the significantly enriched pathways are provided in Table 1.

### 2.4. Patient Samples and Animals

The institutional human ethical committee approval was obtained from Kalinga Institute of Medical Sciences, Bhubaneswar, for collection and use of sera samples from control individuals and IPF patients. Human sera samples from IPF patients (*n* = 10) and healthy individuals (*n* = 20) were acquired. IPF was diagnosed based on the criteria of American Thoracic Society/European Respiratory Society. The demographic details of the patients/healthy individuals are given in Supplementary Table S3. Sera samples were utilized for estimation of sPLA2-IIA. All the samples used in this study are from female individuals with no smoking history. No statistically significant difference was observed between the age distribution of both the groups. The diagnosis of IPF based on the accepted guidelines like the exclusion of known causes of ILD, like environmental exposures both from domestic and occupation, drugs etc., and also by having HRCT patter of UIP.

All mentioned animal experimental protocols in this study were as per CPCSEA (Committee for the Purpose of Control and Supervision of Experiments on animal guidelines), and comply with the ARRIVE guidelines and approved by Animal Ethics Committee, CSIR-Institute of Genomics and Integrative Biology, Delhi, India. C57bl/6 female mice 5–6 weeks old, weighing 18–24 g were acquired from CSIR-Central Drug Research Institute, Lucknow, India and acclimatized for at least 1 week under standard laboratory conditions (25 ± 2 °C, 55% humidity) before starting experiments. We used female mice, since they are less aggressive and do not require care to separate dominant one [29].

### 2.5. Mice Model of Pulmonary Fibrosis

The bleomycin-induced mice model of pulmonary fibrosis is most widely accepted model of pulmonary fibrosis, since it mimics many features of human idiopathic pulmonary fibrosis [30,31]. To determine at which time point after instilling bleomycin fibrosis starts developing, so that we can start giving pBPB, we developed time kinetics mice model of bleomycin-induced pulmonary fibrosis. Wild type female C57bl/6 were randomly divided into five groups: Group 1: Control/VEH (PBS treated mice), Group 2: Bleomycin Day11 (Bleomycin-treated mice, sacrificed on day 11), Group 3: Bleomycin day17 (Bleomycin-treated mice, sacrificed on day 17), Group 4: Bleomycin day21 (Bleomycin-treated mice, sacrificed on day 21) and Group 5: Bleomycin day25 (Bleomycin-treated mice, sacrificed on day 25) (*n* = 4–6 for each group). Bleomycin (Sigma-Aldrich, Saint Louis, MO, USA, cat. No. B2434) was reconstituted in PBS pH 7.4. For the development of lung fibrosis, mice were instilled with bleomycin (1 unit/kg) through orotracheal route on day 0. Mice were first anesthetized by brief isoflurane. After anesthesia, mice were placed on a wood support at an angle of 45 degree, and carefully, the tongue of the mouse was grasped in an upward and leftward position using blunt-end forceps. Now 1 mg/kg of bleomycin was instilled using a pipette. Mice were maintained on same position for 15 sec. and then placed near a warm pad for recovery [32]. Mice were euthanized by xylazine and thiopentone at various time points (Days 11, 17, 21, and 25).

### 2.6. Treatment with pBPB

To observe the effect of pBPB in bleomycin-induced mice model of pulmonary fibrosis, based on results from above time kinetics experiment; we chose most widely used 21 days model of bleomycin-induced pulmonary fibrosis. In this experiment mice were divided into three groups: Group 1. Control/VEH (PBS treated mice), Group 2: Bleomycin/VEH (Bleomycin-treated mice), Group 3: Bleomycin/pBPB (Bleomycin + pBPB treated mice) (*n* = 6 for each group). Mice were anesthetized with isoflurane and bleomycin (1 mg/kg) was instilled via orotracheal route on day 0. pBPB was given orally, twice a day from day12 to bleomycin instilled mice as described in our previous studies. Briefly, pBPB (Sigma) was dissolved in 50% ethanol and given orally 1 mg/kg twice a day from day 12 up to the completion of model.

### 2.7. BAL Fluid Analysis

Mice were anaesthetized and tracheotomized on day 21, 1 mL PBS was instilled into the trachea and recovered PBS (approximately 600 µL) was collected, centrifuged at 1000× *g* to get cell pellets and supernatants; cell pellets were used to count total leucocyte cells as demonstrated by our lab earlier [33]. Supernatant was used to estimate total protein, TGF-β and IL-17 ELISA.

### 2.8. Lung Histopathology and Morphometry

Lungs were harvested, some portion was fixed with 10% formalin, embedded in paraffin, sectioned with microtome and stained with various stains such as hematoxylin and eosin for assessing fibrosis, Masson’s trichrome (MT) staining for observing collagen deposition in lungs. Pulmonary fibrosis scoring was done based on modified Ashcroft scoring, as described by Hubner et al. [34]. Briefly, individual tissue slides were observed in a blindfolded manner and scores were given based on the extent of fibrosis. Images of MT stained slides have been taken and morphometry for quantitation of collagen content was done using Image J software [33,35]. Lung injury were scored based on Semi-quantitative Morphological Index (SMI). Three lung sections from each mouse were observed under light microscope in a blind fold manner and scores were given as described earlier. Briefly, Normal lung: 0; minimal areas of inflammation: 1; more frequent inflammatory areas and fibrosis: 2; all the three sections lung lesions: 3; extensive lesions on at least 2–3 sections: 4; majority of area covered by inflammation and fibrosis: 5 [36,37,38].

### 2.9. Elastin Measurement in Lung Lysate

Total lung lysate was prepared by homogenizing the 50 mg lungs in 500 μL RIPA buffer containing dithiothreitol (Sigma) and protease inhibitor cocktail (Sigma Aldrich, Darmstadt, Germany) containing various protease inhibitors like Aprotinin, AEBSF, E-64, Bestatin, Pepstatin A, and Leupeptin followed by centrifugation. Supernatant was utilized as total lung lysate for estimation of elastin and TGF-β after protein estimation. Elastin was measured using a biochemical assay “Fastin Elastin assay” (Biocolor, Carrickfergus, UK). This assay is based on Fastin dye reagent (5,10,15,20-tertraphenyl-21,23-porphine tetra-sulfonate in a citrate buffer), which binds with elastin present in the sample. Briefly, elastin was first precipitated and dye was added to form an elastin–dye complex. The complex was dissolved by dye dissociation reagent and absorbance was taken at 513 nm wavelength in microplate reader.

### 2.10. sPLA2 Activity Assay

sPLA2 activity was measured in sera of mice as per the manufacture’s instruction (Cayman, Ann Arbor, MI, USA, cat. 765001) with custom modifications. Briefly, the substrate, ethanolic solution of diheptanoyl thio-phosphatidylcholine, was dried under the stream of nitrogen and reconstituted in assay buffer (containing 10 Mm CaCl_2_, 100 Mm of KCl and 0.3 M Triton X-100). DTNB (5, 5′-dithio-bis-(2-nitrobenzoic acid)) added to samples (mice sera 20 μL/assay buffer) or bee venom PLA2, a positive control and in the 96-well plate followed by addition of reconstituted substrate. Absorbance was taken every minute at 414 nm after shaking the plate.

Two time points were selected based on the linear portion of the plot made using absorbance values as a function of time. The following equation was used to determine the change in absorbance:

$${\Delta A}_{414}=\frac{A_{414}\;\left(\mathrm{Time}\;2\right)-{\;A}_{414}\;\left(\mathrm{Time}\;1\right)}{\mathrm{Time}\;2\;\left(\min.\right)-\;\mathrm{Time}\;1\;\left(\min.\right)}$$

$$\mathrm{sPLA}2\;\mathrm{Activity}\;\left(\text{µ}\mathrm{mol}/\min/\mathrm{mL}\right)=\frac{{\Delta A}_{414}/\min.}{10.66{\;\mathrm{mM}}^{-1}}\times \frac{0.130\;\mathrm{mL}}{0.01\;\mathrm{mL}}\times \;\mathrm{Sample}\;\mathrm{dilution}$$

where 10.66 mM^−1^ is the extinction coefficient of DTNB at 414 nm (pathlength = 0.784 cm), 0.130 mL for sera is total volume of the reaction.

### 2.11. ELISA

TGF-β 1 in total lung lysate/BAL fluid (mice), IL-17 in BAL fluid (mice) and sPLA2-IIA in plasma samples of IPF patients and controls were measured using ELISA as per manufacturer’s (ThermoFisher Scientific, Vienna, Austria /Cayman, An Arbor, MO, USA) instructions. Briefly, capture antibody against TGF-β1 or IL-17 or sPLA2-IIA (1:250) was coated in a 96-well plate and kept overnight; it was then washed with 0.05% tween-20 in PBS and blocked with 1% BSA. Then samples (total lung protein or plasma) were added and after 2 h, wells were washed and detection antibody (1:250) was added, followed by a washing step and HRP was added further. After a final wash, the plate was developed with TMB and O.D. was acquired at 450 nm.

### 2.12. Statistics

All the experiments were performed at least three times with six mice per group. All in-vivo data are shown as mean ± SEM and is representative of three independent experiments. All the graphs were generated using GraphPad prism. Two groups and three groups were compared based on unpaired Students *t*-test and one way ANOVA respectively. A *p*-value of 0.05 was considered as significant. In any case, the individual *p* value for each experiment is being mentioned in the respective legend.

## 3. Results

### 3.1. Expression Profiles of Various Secretory Phospholipase A2 Isoforms in IPF Patients and Controls

To study the expression profiles of various secretory phospholipase A2 forms, single cell RNA seq. raw data were acquired from gene expression omnibus [GSE135893, [18]]. The 73,778 single cells from eight control and eight IPF patients were analyzed in the data set A.

Cells were broadly annotated based on the expression of markers and the Seurat object was then split into four subgroups (Supplementary Figure S1A–E):

(a) EPCAM+ epithelial cells (clusters 1 and 4),
(b) PTPRC+ immune cells (clusters 0, 2, 6 and 7),
(c) PECAM1+ and EPAS1+ endothelial cells (cluster 3) and
(d) EPCAM- PTPRC- and PECAM1- mesenchymal cells (cluster 5).

**Figure 1:**
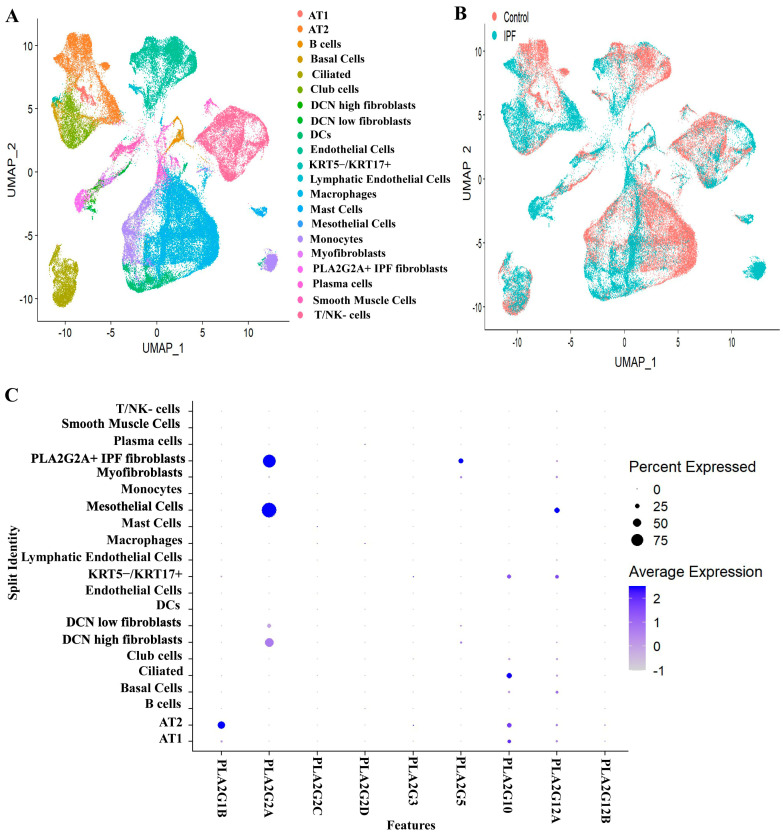
Analysis of single cell RNA seq. data reveals the expression profile of various sPLA2 in data set A (GSE135893). (**A**) Uniform Manifold Approximation and Projection (UMAP) after analyzing and annotating the clusters; Single cell RNA seq data from 8 control (healthy individuals) and 8 IPF patients, using Seurat as described by Habermann et al., showing various cell types. (**B**) UMAP plot showing cells, colored by their origin either from control or IPF patients (**C**) Dot plot showing cellular distribution of PLA2G1B, PLA2G2A, PLA2G2C, PLA2G2D PLA2G3, PLA2G5, PLA2G10, PLA2G12A and PLA2G12B in lung. (Circle size indicates percentage of cells expressing the given gene; intensity of blue color indicates extent of expression).

For each subgroup Seurat object, a similar dimensional reduction, clustering and UMAP visualization approach were applied to get individual UMAP for each subgroup. Each cluster was annotated manually based on the expression of cell type markers. For integrative analysis, all the four annotated subgroup Seurat objects were merged into one large Seurat object followed by SCTransform normalization, scaling, dimensional reduction and UMAP visualization. After merging, using Seurat package in R, we observed 21 cell type identities (annotated based on expression of canonical markers) (Supplementary Figures S2, S7–S9) (Figure 1A), and all these types were coming under 4 major sub-groups like epithelial, immune, endothelial and mesenchymal cells. We observed a relatively novel cell type (PLA2G2A+ IPF fibroblast) that is discussed in a later portion. Among all these compartments, IPF patients have shown a predominant expression of sPLA2 in most of the structural cells like fibroblasts, mesothelial cells, certain types of epithelial cells (ciliated, KRT5−/KRT17+ cells) (Figure 1C). There are 11 isoforms of sPLA2 (IB, IIA, IIC, IID, IIE, IIF, III, V, X, XIIA and XIIB). However, we found prominent expressions of sPLA2-IB (PLA2G1B), sPLA2-IIA (PLA2G2A), sPLA2-III (PLA2G3), sPLA2-V (PLA2G5) sPLA2-X (PLA2G10) and sPLA2-XIIA (PLA2G12A) in the cells present in the lung as shown in Figure 1C, though each type had different cell distribution. In addition to these six isoforms, we observed PLA2G2C, PLA2G2D and PLA2G12B also to be expressed in lungs, but expression was much less, so we have not explored them further. Moreover PLA2G2C is a pseudo gene in humans, while PLA2G12B is expressed in an inactive form [39]. Mesenchymal cells, particularly in fibroblasts, have a predominant expression of PLA2G2A and PLA2G5. While mesothelial cells have a predominant expression of PLA2G2A, epithelial cells have PLA2G1B, PLA2G3, PLA2G10 and PLA2G12A indicating that each cell type might use unique secretory PLA2 isoform for its function (Figure 1C). Interestingly, all these six dominant isoforms were found to be present only in structural cells like fibroblast and epithelial cells that are key players in lung fibrosis. Various immune cells including monocytes, dendritic cells, plasma and B-cells have also shown expressions of PLA2G5 and PLA2G12B, but the percent expression is low (Figure 1C).

### 3.2. Single Cell RNA-seq Analysis of Mesecnymal Cells Reveals PLA2G2A High Fibroblast in IPF Petients

**Figure 2:**
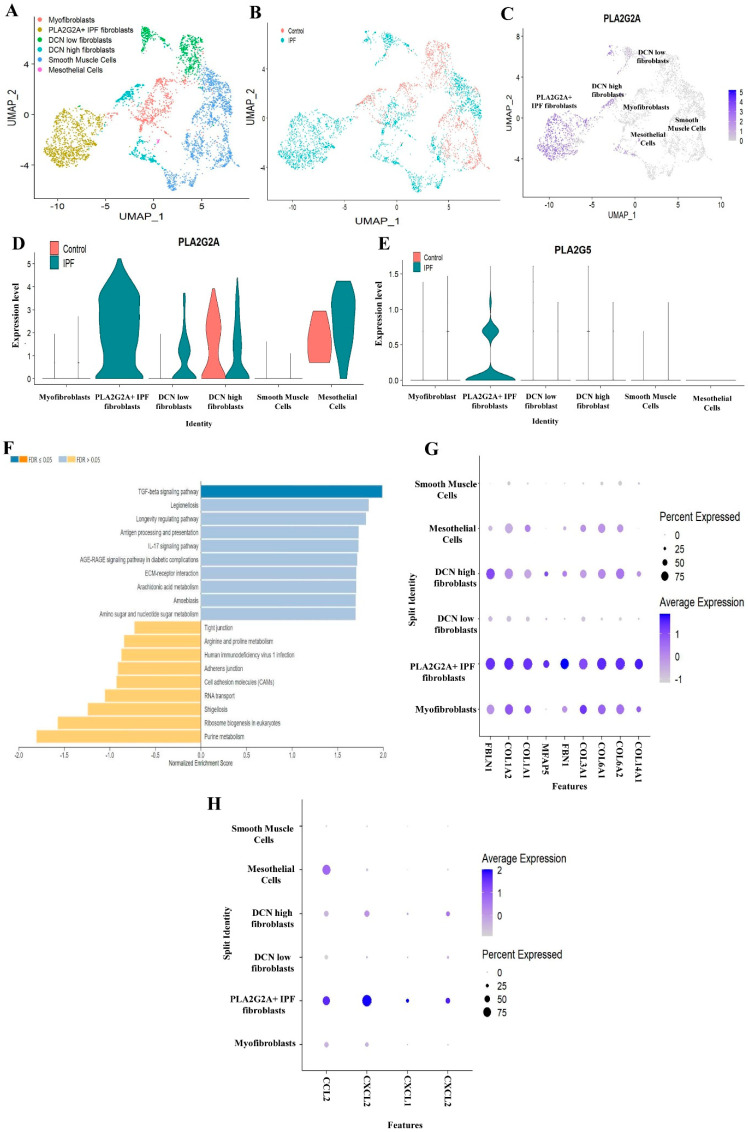
Analyzing expression of PLA2G2A and PLA2G5 in mesenchymal cells in data set A (GSE135893). (**A**) UMAP visualization of mesenchymal cells, various clusters displaying different types of mesenchymal cells. (**B**) UMAP plot showing cells, colored by their origin either from Control or IPF patients. (**C**) Feature plot displaying expression of PLA2G2A. (**D**) Violin plot showing expression of PLA2G2A in various subclusters of mesenchymal cells. (**E**) Violin plot of expression of PLA2G5 in mesenchymal cells. (**F**) Enrichment analysis of pathways differentially regulated in PLA2G2A+ IPF fibroblasts. (**G**) DOT plot showing the upregulation of key ECM genes in mesenchymal cells. (**H**) DOT plot showing the upregulation of key inflammatory genes in mesenchymal cells.

From the above results, it is clear that PLA2G2A is expressed in mesenchymal cells, particularly in fibroblasts and mesothelial cells whereas PLA2G5 is expressed in fibroblasts. However, we wanted to study the expressions of PLA2G2A and PLA2G5 at the single cell level in the mesenchymal cell subgroup Seurat object of IPF patients compared to controls. We observed six sub clusters after re-analysis of mesenchymal cells subgroup Seurat object (Figure 2A). Based on known markers and expression of signature genes, these clusters are annotated as (a) DCN (Decorin) high fibroblast [LUM+ (lumican, a proteoglycan) and DCN high cells), (b) DCN low fibroblast (LUM+ and DCN low cells), (c) Myofibroblast [LUM and ACTA2+ (smooth muscle α actin) cells], (d) PLA2G2A IPF fibroblasts (LUM and PLA2G2A high cells), (e) smooth muscle cells (ACTA2+ cells) and (f) mesothelial cells (UPK3B+ and CALB2+) (Supplementary Figure S2A–E). During annotation, we identified a sub-cluster of fibroblasts highly expressing PLA2G2A, and this population is specific to IPF patients only (Figure 2B–D). Since PLA2G2A was the most upregulated gene in this population, and this population is specific to IPF patients, we named it as PLA2G2A+ IPF fibroblasts (Figure 2A–D). To study the expression of both PLA2G2A (Figure 2D) and PLA2G5 (Figure 2E) in mesenchymal cells, a violin plot was generated. We found high expression of PLA2G2A and PLA2G5 in PLA2G2A+ IPF fibroblasts. In addition to fibroblasts, mesothelial cells have also shown a high expression of PLA2G2A in IPF patients (Figure 2D–E).

The pathway enrichment analysis revealed a significant upregulation of TGF-β signaling pathway, IL−17 signaling, arachidonic acid metabolism pathway, ECM-receptor interaction and other fibrosis-related pathways indicating the active role of PLA2G2A+ IPF fibroblasts in IPF (Figure 2F and Table 1). In addition to this, analysis of top 100 genes that are differentially regulated in PLA2G2A+ IPF fibroblasts has shown upregulation of multiple key extra cellular matrix (ECM) genes such FBLN1, COL1A2, COL1A1, MFAP5, FBN1, COL3A1, COL6A1, COL6A2 and COL14A1 (Figure 2G). Moreover, a number of chemokines and cytokines involved in inflammation including CCL2, CXCL12, CXCL1 and CXCL2 are upregulated in PLA2G2A+ IPF fibroblasts (Figure 2H). This indicates that PLA2G2A+ IPF fibroblasts has both inflammatory and fibrotic properties.

**Figure 3:**
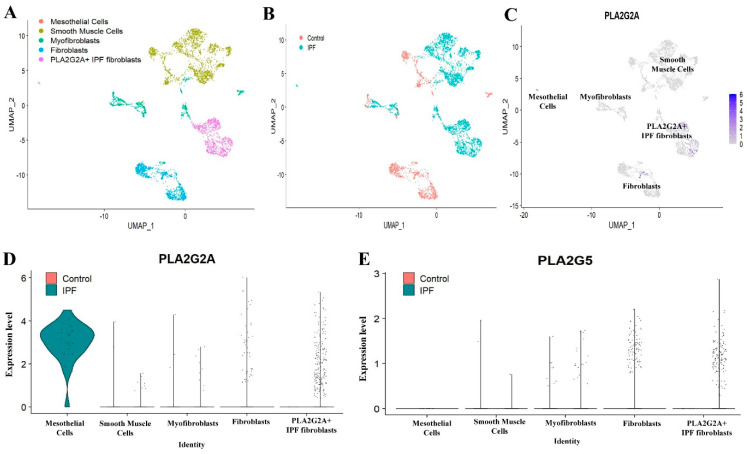
Analyzing expression of PLA2G2A and PLA2G5 in mesenchymal cells in data set B (GSE132771). (**A**) UMAP visualization of mesenchymal cells, various clusters displaying different types of mesenchymal cells. (**B**) UMAP plot showing cells, colored by their origin either from Control or IPF patients. (**C**) Feature plot displaying expression of PLA2G2A. (**D**) Violin plot showing expression of PLA2G2A in various subclusters of mesenchymal cells. (**E**) Violin plot of expression of PLA2G5 in mesenchymal cells.

We wanted to validate the existence of PLA2G2A+ IPF fibroblast in other datasets that are publicly available as mentioned in Methods. In the second dataset (dataset B, GSE132771) [19], we studied the expressions of PLA2G2A and PLA2G5 in the mesenchymal cells of IPF patients compared to controls. Particularly, mesenchymal cells are extracted based on the markers of various mesenchymal cells from all the lung cells (Supplementary Figure S3A–D) Based on known markers and expression of signature genes, these clusters are annotated as (a) UPK3B+ and CALB2+ mesothelial cells; (b) ACTA2+ smooth muscle cells (c) LUM+ and ACTA2+ Myofibroblasts, (d) DCN+ and LUM+ fibroblasts and (e) LUM+ PLA2G2A high “PLA2G2A IPF fibroblasts” (Supplementary Figure S4A–E). We observed five sub clusters (Figure 3A). Similar to first dataset, this dataset had also shown “PLA2G2A IPF fibroblasts” almost exclusively in IPF patients. Even though mesothelial cells are very scarce, IPF patients had shown dominant expression of PLA2G2A in mesothelial cells compared to controls (Figure 3B–D). PLA2G5 (Figure 3E) is also found to be expressed in PLA2G2A IPF fibroblasts in IPF patients.

**Figure 4:**
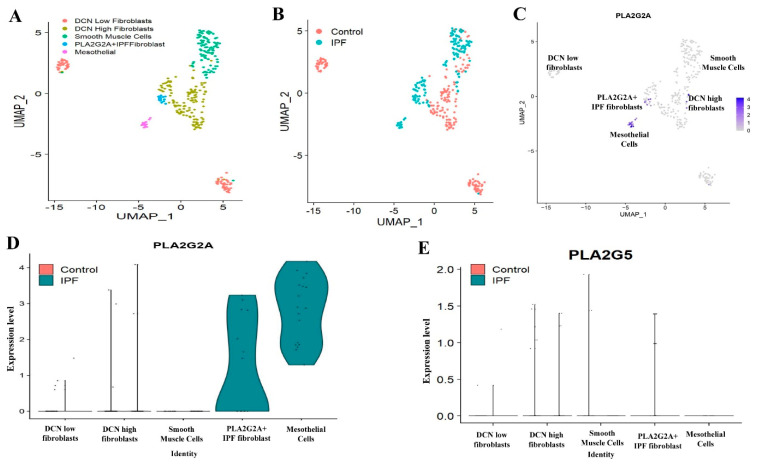
Analyzing expression of PLA2G2A and PLA2G5 in mesenchymal cells in data set C (GSE122960). (**A**) UMAP visualization of mesenchymal cells, various clusters displaying different types of mesenchymal cells. (**B**) UMAP plot showing cells, colored by their origin either from Control or IPF patients. (**C**)Feature plot displaying expression of PLA2G2A. (**D**) Violin plot showing expression of PLA2G2A in various subclusters of mesenchymal cells. (**E**) Violin plot of expression of PLA2G5 in mesenchymal cells.

In the third dataset (dataset C, GSE122960) [20] also, we extracted mesenchymal cells (Supplementary Figure S5A–D) and found five sub-clusters like DCN low fibroblasts, DCN high fibroblasts, smooth muscle cells, PLA2G2A IPF fibroblasts and mesothelial cells (Figure 4A, and Supplementary Figure S6A–E). Both “PLA2G2A IPF fibroblasts” and mesothelial cells had shown dominant expression of PLA2G2A in IPF patients compared to controls (Figure 4B–D), whereas PLA2G5 (Figure 4E) is relatively scarce compared to earlier datasets.

### 3.3. PLA2G1B, PLA2G3 PLA2G10 and PLA2G12A Isoforms Have Shown Differential Expression in IPF Patients

**Figure 5:**
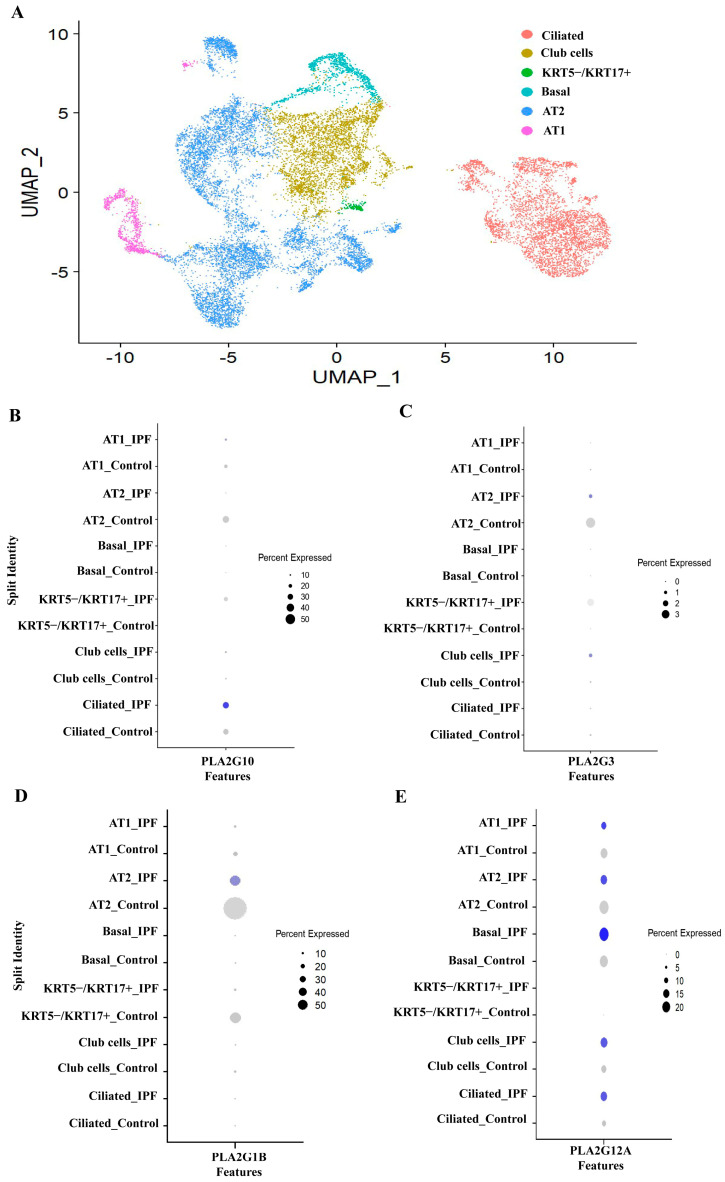
Analyzing expression of PLA2G10 and PLA2G3 in epithelial cells (**A**) UMAP showing the distribution of various clusters of epithelial cells. (**B**–**E**) Dot plot visualization of expression of PLA2G10, PLA2G3, PLA2G1B and PLA2G12A in epithelial cells from Control and IPF patients.

We found expression of PLA2G1B, PLA2G3, PLA2G10 and PLA2G12A in epithelial cells, therefore the differential expression of PLA2G1B, PLA2G3,PLA2G10 and PLA2G12A were assessed in epithelial cells by dot plot. Further sub-clustering of epithelial cells results in six sub-clusters (Figure 5A). Based on known markers and expression of signature genes, these clusters are annotated as Club cells [secretoglobin family 1A member 1 (SCGB1A1) + and secretoglobin family 3A member 1 (SCGB3A1)+)], Ciliated cells [Forkhead Box J1 (FOXJ1+)], AT1 cells [advanced glycosylation end-product specific receptor (AGER)+ alveolar type 1 cells), Basal cells [Keratin 5 (KRT5) + and KRT17+ cells), AT2 cells [surfactant protein-C (SFTPC)+ alveolar type 2 cells] and KRT5−/KRT17+ (KRT5 negative and KRT17+ cells) (Supplementary Figure S7A–G). The KRT5−/KRT17+ cells are one of the recently identified epithelial population having pro-fibrotic roles [18,40]. PLA2G10 was found to be increased both in ciliated cells and KRT5−/KRT17+ cells in IPF individuals while AT2 has shown reduction in expression of PLA2G10 (Figure 5B). We observed increased expression of PLA2G3 in club cells and KRT5−/KRT17+ cells in IPF individuals as compared to control (Figure 5C). Expression of PLA2G1B was found to be decreased in AT cells of IPF patients (Figure 5D). PLA2G12A has shown increased expression in club and ciliated cells, while it was reduced in AT1 and AT2 cells in IPF patients (Figure 5E) as compared to healthy individuals.

### 3.4. Expression of sPLA2-IIA Increased in the Plasma Samples of IPF Patients

**Figure 6:**
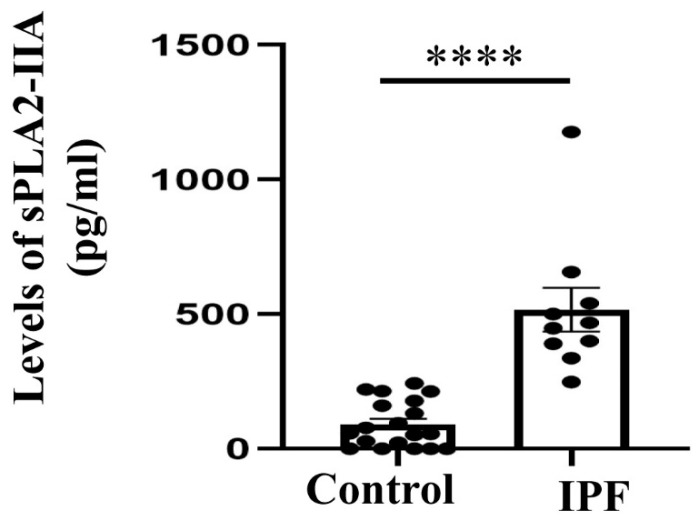
sPLA2-IIA levels are found to be increased in human IPF patients. ELISA that measures sPLA2-IIA in human sera of both IPF patients and healthy controls. (Values are expressed in means ± SEM, **** *p* value = 0.0001).

We selected sPLA2-IIA for validation of single cell RNA seq data analysis, as we found predominant and increased expression of PLA2G2A (sPLA2-IIA) in IPF patients as compared to healthy individuals. There were 20 healthy controls and 10 IPF patients (Supplementary Table S3), and we have measured sPLA2-IIA in sera of both controls and patients by ELISA as mentioned in Methods. We found elevated levels of sPLA2-IIA in the sera of IPF patients as compared to healthy individuals (Figure 6A).

### 3.5. Development of Bleomycin-Induced Pulmonary Fibrosis with Time Kinetics

**Figure 7:**
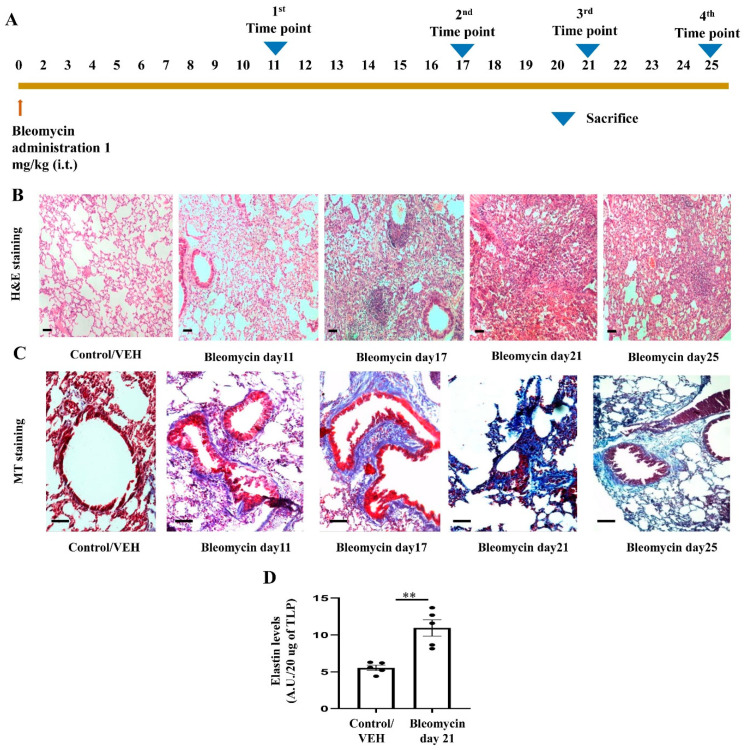
Development of mice model of bleomycin-induced pulmonary fibrosis (**A**) Experimental protocol for developing mice model of pulmonary fibrosis. (**B**) H & E staining (bars = 100 µm) showing the morphology of lungs from control and bleomycin-treated mice. (**C**) MT staining (bars = 100 µm) depicting collagen deposition in lungs of control and bleomycin-treated mice. (**D**) Elastin assay in total lung lysate from control and bleomycin-treated mice. ** *p* = 0.003.

We found increased expression of various sPLA2 in IPF patients as compared to healthy individuals while most of the sPLA2 has a pro-inflammatory role and the end stage of a number of inflammatory conditions is fibrosis [41,42,43]. Therefore, we hypothesized that sPLA2 could be a potential therapeutic target for pulmonary fibrosis. Earlier, we showed that pBPB attenuates certain features of both acute as well as chronic asthma in the Ova-induced mice model. We have shown that pBPB reduced sub-epithelial fibrosis and reduced levels of TGF-β in mice model of allergic asthma [21,22]. On the other hand, pBPB is PLA2 inhibitor and PLA2 may activate fibrosis via the arginase/TGF-β pathway [21,22], therefore we hypothesized that pBPB may attenuate certain features of pulmonary fibrosis. The bleomycin mice model is a widely accepted model for lung fibrosis, mimicking many features of idiopathic pulmonary fibrosis. To find out at which time point fibrosis starts developing so that we can start giving pBPB, we did a time kinetics study in which we have instilled bleomycin to mice and sacrificed mice at various time points as shown in Figure 7A. We found that after day11 of bleomycin instillation, inflammation started resolving and fibrosis started developing. To validate whether bleomycin-treated mice have developed fibrosis, we performed various assays. We performed H and E staining to observe the morphology of lung section. From day11, alveolar disruption starts appearing, though we could observe fibrotic loci after day17 time point (Figure 7B). Collagen deposition is one of the hallmark features of pulmonary fibrosis. To observe collagen deposition, we performed Masson’s trichrome staining, in which collagen is stained by aniline blue stain. In the early time point i.e., from Day 11 only, we observed collagen deposition in the lungs of bleomycin-treated mice (Figure 7C). Since elastin fibers are also accumulated in the lungs of bleomycin-induced fibrosis mice, so to assess the same, we performed a fastin elastin assay in Day 21 groups and controls and found that elastin levels are upregulated as compared to control mice (Figure 7D). We found that at all the time points starting from day 11, most of the features of pulmonary fibrosis are upregulated as compared to the control, while day 21 is the most widely accepted model, so we used the same in this study.

### 3.6. pBPB Attenuates Pulmonary Fibrosis in Bleomycin-Induced Mice

**Figure 8:**
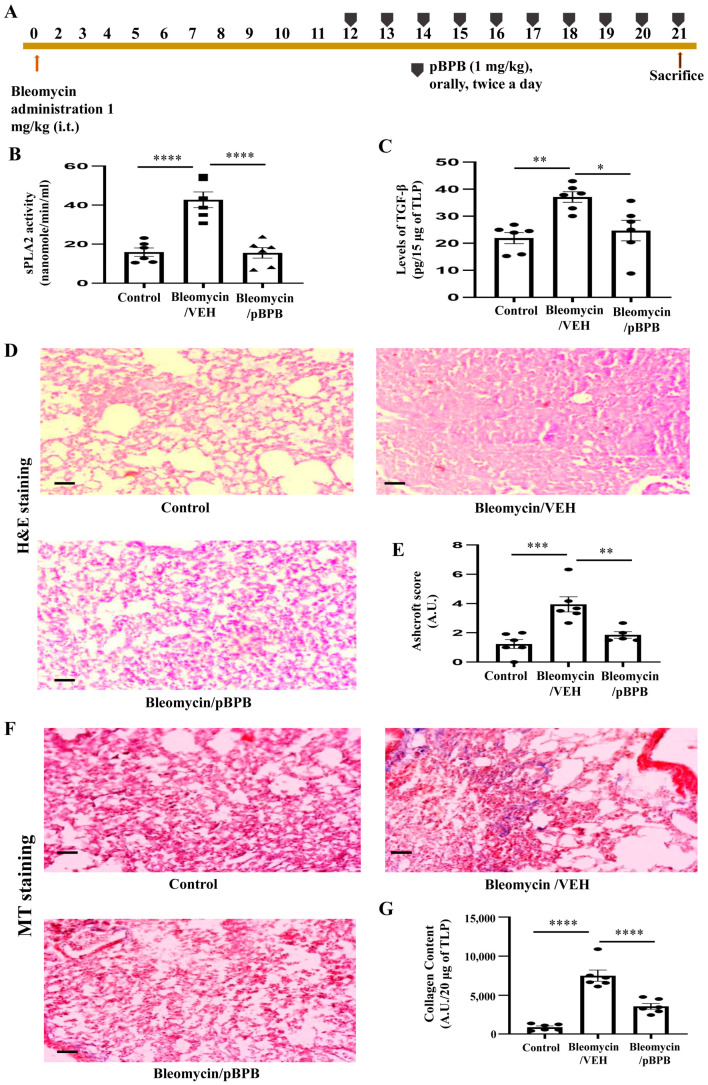
pBPB ameliorates pulmonary fibrosis in Bleomycin-treated mice (**A**) Schematic showing the mice experimental plan, mice were treated with bleomycin (orotracheal instillation) and from day 12 onwards, pBPB was given orally; twice a day and after euthanasia, mice were sacrificed on day 21. There were three groups and *n* = 6 in each group. (**B**) sPLA2 activity and (**C**) TGF-β1 ELISA was performed in mice sera samples and total lung lysates of bleomycin-treated mice, respectively, **** *p* = 0.0001 for control versus Bleomycin/VEH group and **** *p* = 0.0001 for Bleomycin/VEH versus Bleomycin/pBPB group, and ** *p* = 0.003 for control versus Bleomycin/VEH group, * *p* = 0.014 for Bleomycin/VEH versus Bleomycin/pBPB group,sPLA2 activity and TGF-β1, respectively. (**D**) and (**E**) H and E staining (bars = 100 µm) revealed attenuation in pulmonary inflammation in Bleomycin/pBPB treated mice, Ashcroft score. *** *p* = 0.0004 for control versus Bleomycin/VEH group and ** *p* = 0.0056 for Bleomycin/VEH versus Bleomycin/pBPB group (**F**) and (**G**) MT staining (bars = 100 µm) results has shown reduced collagen deposition in lungs of Bleomycin/pBPB treated mice as compared to Bleomycin/VEH mice, semi-quantitative morphometry to assess collagen deposition in lungs. *n* = 6. **** *p* = 0.0001 for control versus Bleomycin/VEH group and **** *p* = 0.0001 for Bleomycin/VEH versus Bleomycin/pBPB group.

As we found increased expression of PLA2-IIA in the sera of IPF patients and increased activity of sPLA2 in lungs of bleomycin-treated mice, we wanted to study the effect of inhibition of sPLA2 by pBPB. Bleomycin-administered mice were treated with pBPB (orally, 1 mg/kg, twice a day) from Day 12–Day 21 as shown in Figure 8A. The pBPB treatment reduced the activity of sPLA2 (Figure 8B). TGF-β plays a vital role in pathogenesis of pulmonary fibrosis, therefore we assessed levels of the same. TGF-β was found to be reduced in Bleomycin/pBPB-treated mice as compared to only Bleomycin/VEH-treated mice (Figure 8C). Then we performed hematoxylin and eosin staining in the formalin fixed lung sections. As shown in Figure 8D, pBPB treatment reduces fibrotic loci as compared to only bleomycin-treated mice. The semi-quantitative measurement of fibrosis by modified Ashcroft scoring suggested reduced fibrosis in Bleomycin/pBPB-treated mice as compared to only Bleomycin/VEH-treated mice. (Figure 8E). The lung injury score by Semi-quantitative Morphological Index further supported the above findings (Supplementary Figure S10). We wanted to determine whether pBPB reduces collagen deposition in the lungs of bleomycin-treated mice by performing MT staining. In bleomycin-treated mice, collagen deposition was observed, but in Bleomycin/pBPB treated mice, collagen deposition was found to be reduced as compared to only bleomycin-treated mice (Figure 8F,G). Further BAL fluid analysis has shown reduction in total protein content and infiltration of inflammatory cells in BAL fluid of Bleomycin/pBPB treated mice as compared to Bleomycin/VEH treated mice (Supplementary Figure S11A,B). We found decreased levels of inflammatory cytokine, IL-17 and pro-fibrotic cytokine; TGF-β in BAL fluid of Bleomycin/pBPB treated mice as compared to Bleomycin/VEH treated mice (Supplementary Figure S11C,D).

## 4. Discussion

The entire PLA2 superfamily has more than 30 members; among them, there are 11 isoforms of sPLA2 (IB, IIA, IIC, IID, IIE, IIF, III, V, X, XIIA and XIIB) [7]. However, we found predominant expression of PLA2G1B, PLA2G2A, PLA2G3, PLA2G5, PLA2G10 and PAL2G12A in the cells present in the lung as shown in Figure 1C, though each type had different cellular distribution. This differential cellular distribution and known differential selectivity of each of these isoforms indicate that each sPLA2 seems to have distinct pathophysiological role [39]. The sPLA2 isoforms have been shown to have an important role in regulation of inflammation, remodeling of membrane and also digesting exogenous phospholipids present in food and microbes. Interestingly, all these six dominant isoforms were found to be present only in structural cells like fibroblasts (PLA2G2A and PLA2G5), mesothelial cells (PLA2G2A) and epithelial cells (PLA2G1B, PLA2G3, PLA2G10 and PLA2G12A) that are key players in lung fibrosis. All these findings indicate the possible innate immune role of sPLA2 secreted by fibroblasts mesothelial cells and epithelial cells.

In this regard, we found relatively unique and specific expression of PLA2G2A in a subset of fibroblast (PLA2G2A+ IPF fibroblast) that is very specific to IPF (Figure 2A–D) and also validated the same in two different single cell-RNA seq data sets (Figure 3A–E and Figure 4A–E). We do not know the biological significance of this PLA2G2A high fibroblast in IPF patients. Compared to other sPLA2 isoforms, sPLA2-IIA (PLA2G2A) is only found to be increased in the circulation [39], and sPLA2-IIA is otherwise called “bactericidal” or “inflammatory” PLA2 as it is has substrate specificity on phosphatidylethanolamine that is mostly found in the bacterial membranes and has a high cationic property to cause hydrolysis of the bacterial membrane. In addition to acting on foreign microbes, sPLA2-IIA also acts on membranes of extracellular mitochondria that are released at the site of inflammation either from leukocytes or platelets. Upon being targeted by sPLA2-IIA, these mitochondria act on neutrophils that further adhere to the vessel walls and this step is crucial in innate immune mechanisms [39,44]. In this context, it is well demonstrated that neutrophil and its components promote not only lung inflammation but also initiate fibrogenesis in various chronic lung diseases including IPF [45].

Further, sPLA2-IIA has been shown to be linked to the degradation of surfactant proteins that are rich in phosphatidylglycerol, which is one of the favorable substrate for sPLA2-IIA [7]. This indicates that sPLA2-IIA can cause lung collapse. Evidently, a number of studies have demonstrated increased levels of sPLA2-IIA in various secretions like BAL fluid and the sera of patients with acute respiratory distress syndrome [7], severe COVID-19 patients [46], and also in alveolar fluids of patients with IPF and bleomycin lung (lungs of patients who had been administered bleomycin as a therapy) [47]. The significantly elevated levels of sPLA2-IIA in the plasma of COVID-19 patients were found to be well correlated with mortality [46]. Further, they have identified sPLA2-IIA as one of the critical variables among the 80 indices for predicting COVID-19 mortality. In the present study, we found an elevated level of sPLA2-IIA in IPF patients compared to healthy individuals. There are some limitations in our study. All patients are females and some of the patients are younger. It is known that IPF mostly affects older individuals. However, multiple studies had found that IPF may affect young individuals [48,49,50,51,52]. Leuschner et al. has reviewed the medical records from February 2011 to February 2015, and they found that out of 440 patients with interstitial lung diseases (ILD), 23% patients were IPF with an age group of ≤50 years [29]. Nadrous et al. had shown that the younger IPF patients (range 28–49 years) had the same poor prognosis as the old patients [31]. Study by Ganesh Raghu et al. had shown incidence and prevalence of IPF in younger IPF patients, though they have shown reduced in younger patients [32]. The reason behind IPF in younger individuals may be familial [52].

**Figure 9:**
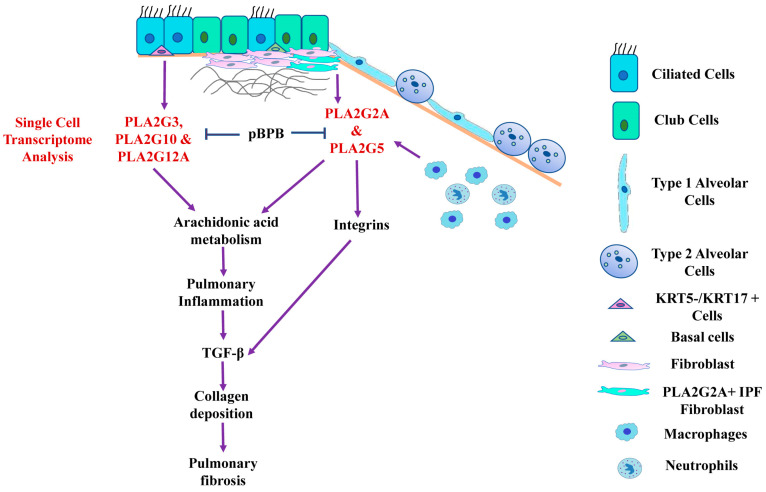
Schematic diagram showing the cellular distribution and possible role of sPLA2 in IPF patients and its inhibition in Bleomycin-induced mice. Single-cell transcriptome analysis revealed increased expression of PLA2G2A and PLA2G5 in fibroblasts, PLA2G10 in KRT5−/KRT17+ cells and ciliated cells, PLA2G3 in KRT5−/KRT17+ cells and club cells and PLA2G12A in club cells and ciliated cells of IPF patients. PLA2G2A and PLA2G5 can interact and activate integrins, which in turn activate TGF-β, the master regulator of pulmonary fibrosis. sPLA2 is involved in arachidonic acid metabolism and resulting metabolites can drive pulmonary inflammation and fibrosis. pBPB inhibit sPLA2 and attenuate certain features of fibrosis including collagen deposition and TGF-β in bleomycin-treated mice.

sPLA2-IIA has been shown to stimulate cardiac fibroblasts to transdifferentiate into myofibroblasts through EGFR activation [11]. However, the pro-fibrotic role of sPLA2-IIA has not been demonstrated in lung fibrosis. sPLA2-IIA is also secreted by synovial fibroblasts of rheumatoid arthritis, and can amplify the synovial inflammation [53]. In addition to this, sPLA2-IIA converts mononuclear cells present in the atherosclerotic plaques into aggressive cells with increased adhesion and migration properties along with the characters of monocyte-derived dendritic cells. These findings indicate that sPLA2-IIA can act as a link between innate and acquired immunity to accelerate the atherosclerosis-related complications [54]. We also found upregulation of both inflammation- and fibrosis-related pathways in PLA2G2A+ IPF fibroblast in IPF like the TGF-β signaling pathway, IL-17 signaling, the arachidonic acid metabolism pathway and the ECM-receptor interaction (Figure 2F and Table 1). Multiple ECM genes are significantly upregulated in PLA2G2A+ fibroblasts (Figure 2G); this indicates that the fibroblast population may be crucial for the excessive deposition of extracellular matrix in the fibrotic lungs. All these findings indicate that the PLA2G2A+ fibroblasts population in IPF may have both fibrotic and inflammatory properties. In general, fibroblasts are not considered as active immune cells, though it is well known that its activation during tissue injury is strongly associated with the fibrosis of the concerned tissue/organ. The possible reason for such ignorance seems to be non-availability of specific markers for the fibroblasts. However, single-cell studies indicate that fibroblasts are like a heterogeneous sub-population of a variety of cells like mesenchymal stromal cells, pericytes etc. Thus, the activation of these fibroblasts with more proliferation and the secretion of various pro-inflammatory mediators including sPLA2-IIA convert these fibroblasts into aggressive inflammatory fibroblasts that cross-talk with various immune cells to precipitate the fibrosis. Thus, it has been suggested that identification of such markers that are behind such transformation could lead to very focused therapeutic strategies to treat fibro-proliferative and fibro-contractive diseases. As sPLA2-IIA seems to have such property of changing the harmless fibroblasts to more inflammatory fibroblasts, targeting this could be beneficial in lung fibrosis [55]. Multiple studies have shown that sPLA2-IIA can interact and activate integrins [56,57,58]. While integrins are well known for their role in activation of TGF-β and fibroblast migration in pulmonary fibrosis, this could be the possible mechanism by which sPLA2-IIA can participate in the pathogenesis of pulmonary fibrosis [59,60,61,62] (Figure 9).

We found increased expression of PLA2G5 in PLA2G2A+ IPF fibroblast population (Figure 2E and Figure 3E) and this increased PLA2G5 can target PC (Phosphatidylcholine) and surfactant proteins present in plasma membrane. PLA2G5 is less-studied compared to PLA2G2A; it has an inflammatory role via arachidonic acid mobilization and eicosanoid generation [7]. It has greater affinity to bind to the phosphatidyl choline of the plasma membrane and pulmonary surfactant, thus it can hydrolyze lung surfactant [7], while the surfactant disruption can cause alveolar injury and thus participate in the pathogenesis of IPF [63]. Thus, our study further supports the hypothesis that sPLA2s, like PLA2G2A and PLA2G5, may act as a prognostic marker in IPF. sPLA2-V is otherwise called Th2-prone sPLA2 due to its nature of potentiating allergic airway inflammation. The decreased expression of IL-4 and IgE synthesis in PLA2G5 knockout mice indicates its dominant role in Th2-mediated immunity. In addition to its expression in Th2 cells, it is also found to be expressed in airway epithelial cells and thus is involved in the promotion of airway injury with degradation of surfactants [39]. In general, the Th2 immune response is known to coordinate the tissue repair and fibrosis through the involvement of multiple immune and non-immune cells types including fibroblasts and epithelial cells.

A number of studies have indicated that airway epithelium act as an innate cell as it has to face the external environment, and the innate immune role of fibroblasts might be interesting as they are located just beneath the basement membrane and away from the external environment compared to epithelial cells. More investigations are required to find the possible mechanisms for the upregulation of sPLA2-IIA in IPF patients and to see whether sPLA2-IIA can be taken as an effective therapeutic target to prevent the lung fibrosis.

KRT5−/KRT17+ cells are recently identified basal-like epithelial cell phenotype, highly enriched in IPF patients and known for their role in ECM production [18,40]. Accumulating evidences suggested that both the club cells and KRT5−/KRT17+ cells are known to play a pro-fibrotic role [18,64,65], and we also found an increased expression of PLA2G3 in club cells and KRT5−/KRT17+ cells of IPF patients as compared to control and healthy individuals, which further supports the same hypothesis. PLA2G10 is also found to be increased in ciliated cell and KRT5−/KRT17+ cells in IPF patients as compared to control healthy individuals, indicating the pro-fibrotic role of these cells in IPF [18,66]. Expression of PLA2G12A was found to be increased in club and ciliated cells of IPF patients as compared to control healthy individuals. However, decreased expression of PLA2G10, PLA2G3, PLA2G1B and PLA2G12A in AT1 and AT2 cells of IPF patients compared to controls indicated that different isoforms may have different disease modulatory roles. In fact, it has been suggested that each sPLA2 isoform is having tissue specific roles as it acts on a variety of specific phospholipid substrates (34). Multiple studies have observed the hydrolysis of surfactant phospholipids by various isoforms of sPLA2 (like PLA2G1B, PLA2G2A, PLA2G5 and PLA2G10) [63,67,68]. While surfactant protein-B (SP-B), produced by AT2 cells, is known to inhibit hydrolysis of surfactant phospholipids by sPLA2, AT2 hyperplasia is the key pathological feature of pulmonary fibrosis [69]. Thus, one can expect more synthesis of SP-B in pulmonary fibrosis. Moreover, increased surfactant may lead to impaired lung function during pulmonary fibrosis [63]These also indicate the need for identification of isoform specific inhibitors for effective therapeutic strategies.

sPLA2-X is also considered as asthmatic sPLA2 (34). It has been demonstrated that sPLA2-X is secreted by airway epithelium, and further, this released sPLA2-X acts on the phospholipids present in the immune cells like eosinophils and releases lysophospholipid, thus increasing the generation of CysLT from eosinophils. Importantly, these entire events are inhibited by inhibition of cPLA2 alpha [70]. These indicate that sPLA2 seems to have a dominant role compared to cPLA2alpha, and on the other hand, it seems that structural cells like airway epithelia augment airway inflammation through sPLA2 [70,71,72].

The physiological function of PLA2G12A is not clear yet, though it is highly expressed in various tissues. It is both structurally and functionally different from other sPLA2 and has shown much less sPLA2 activity [39]. A genome RNA expression profiling study had shown upregulation of PLA2G12A in addition to PLA2G1B and PLA2G2D in patients with pulmonary arterial hypertension secondary to IPF [73].

We found increased expression of PLA2G2D in macrophages and ciliated cells in IPF patients (Figure 1C), indicating its innate immune role while earlier literature had shown polymorphism in PLA2G2D is co-related with body weight loss in COPD patients [39]. However further studies are required to demonstrate the role of PLA2G2D in lung diseases.

Thus, sPLA2 seems to have a disease-associated role in IPF. Next, we wanted to determine the possible therapeutic effect of sPLA2 in the human-relevant mice model of IPF. The American Thoracic Society recommends the use of male mice to develop Bleomycin-induced lung fibrosis. However, the NIH recommends the use of both male and female mice to develop Bleomycin-induced lung fibrosis. In any event, a number of studies had used female mice for developing Bleomycin-induced lung fibrosis [74,75,76,77,78]. For example, study by Ruscitti et al. had shown the assessment of lung fibrosis by Micro-CT correlates with the histological manifestation of fibrosis in bleomycin-induced female mice [74]. Gharaee-Kermani et al. [79] demonstrated that female mice are having exaggerated lung fibrotic response compared to male animals in bleomycin model. They further demonstrated the reduced fibrosis in the ovariectomized, bleomycin-treated rats whereas estradiol replacement restored the lung fibrotic response that was comparable to intact female mice. In the present study, we also observed that C57BL/6 female mice develop multiple features of pulmonary fibrosis (Figure 7).

While there is always a debate regarding the role of animal models in human translational approaches, it will be ideal to explore the pathways that are common to both human and animal models. In this context, a number of commonly expressed genes were found upon comparison between human lung fibrotic condition and the rodent model of bleomycin-induced lung fibrosis, indicating that these genes can be taken as a target in human-translational strategies. One such common gene is PLA2G2A, which was found to be upregulated significantly in the lungs of both rodent and human fibrotic conditions [80]. pBPB is a known sPLA2 inhibitor, and in our previous studies, we have shown the anti-asthmatic role of pBPB along with reduction in sub-epithelial fibrosis [21,22]. Though we have selected pBPB based on our earlier findings in the asthma study [21,22], there are newer pharmaceutical agents like varespladib (LY315920) and its prodrug methyl-varespladib (LY333013) that had shown very effective sPLA2 inhibition even at nanomolar concentrations [81,82], which should be explored for their anti-fibrotic activities in lung fibrosis in future. It has been demonstrated that pBPB alkylates the histidine residue in the active site of sPLA2 to inactivate it without affecting the cPLA2 activation [16,83,84]. However, there is no report to demonstrate its effect on specific isoforms of sPLA2.

There is a considerable overlap between asthma and lung fibrosis in the context of sPLA2. Arachidonic acid metabolites such as leukotrienes and thromboxane are increased in both the diseases asthma and lung fibrosis and are well known for their pro-inflammatory roles. Our previous study has shown that pBPB; a known sPLA2 inhibitor attenuated asthma along with reduction in sub-epithelial fibrosis in a mice model [21,22]. We have chosen pBPB, a known sPLA2 inhibitor, in this present study. We have observed that pBPB treatment reduces pulmonary inflammation, extracellular matrix deposition (collagen) and TGF-β in bleomycin-treated mice (Figure 8, Supplementary Figure S10 and S11), which indicates the possible therapeutic role for pBPB in pulmonary fibrosis. Though we have focused on sPLA2, pBPB is a nonspecific inhibitor of PLA2 as it inhibits a number of enzymes such as phospholipase C and GSK-3β [85]. The GSK-3β is also a serine/threonine kinase, and its signaling is found to be essential in the conversion of pulmonary fibroblasts into myofibroblasts [85]. The inhibition of GSK-3 also has been shown to reduce the progression of pulmonary fibrosis [86]. It is difficult to conclude whether the pBPB mediated effects are only by inhibiting sPLA2. In any case, there is a need for a very specific inhibitor for sPLA2-IIA. More investigations are required to find the sPLA2-IIA-specific inhibitor along with more in-depth characterization of the PLA2G2A high-fibroblast population in IPF patients.

## 5. Conclusions

In summary, we have revealed the PLA2G2A high fibroblast population with both fibrotic and inflammatory properties in the lungs of IPF patients. Expression of PLA2G2A and PLA2G5 were found to be increased in fibroblasts of IPF patients whereas PLA2G3, PLA2G10 and PLA2G12A were increased in certain key epithelial cells like club cells, KRT5−/KRT17+ cells and ciliated cells (Figure 9). In addition to this, we found elevated sera levels of sPLA2-IIA in IPF patients. Further, inhibition of sPLA2 by pBPB ameliorates pulmonary fibrosis in bleomycin-induced mice (Figure 9).

## Figures and Tables

**Table 1:** The significantly enriched pathways in PLA2G2A+ IPF fibroblasts.

S. No.	Pathway	Normalized Enrichment Score	*p*-Value
1.	TGF-beta signaling pathway	1.9917	<2.2 ×10^−16^
2.	Longevity regulating pathway	1.8124	<2.2 × 10^−16^
3.	Legionellosis	1.8419	0.0011287
4.	AGE-RAGE signaling pathway in diabetic complications	1.7161	0.0063559
5.	IL-17 signaling pathway	1.7311	0.0066445
6.	Antigen processing and presentation	1.7329	0.0081112
7.	ECM-receptor interaction	1.7329	0.0086674
8.	Amino sugar and nucleotide sugar metabolism	1.6986	0.0093085
9.	Arachidonic acid metabolism	1.7057	0.010127
10.	Amoebiasis	1.7010	0.011737

## Data Availability

All the data is available either in manuscript or supplementary files.

## Supplementary Materials

The following supporting information can be downloaded at: https://www.mdpi.com/article/10.3390/cells12071044/s1, Supplementary Table S1. Patient’s demographic data as described by Habermann et al. in original publication. Supplementary Table S2. Patient’s demographic data as described by Reyfman et al. in original publication. Supplementary Table S3. Demographic profile of patients and healthy individuals utilized for measuring sPLA2-IIA in human sera of both IPF patients and controls. Supplementary Figure S1. Analysis of single cell RNA seq. data from data set A to show the various cellular compartments as clusters. Supplementary Figure S2. Feature plot displaying the expression of markers for various types of mesenchymal cells in data set A. Supplementary Figure S3. Analysis of single cell RNA seq. data from data set B to show the various cellular compartments as clusters. Supplementary Figure S4. Feature plot displaying the expression of markers for various types of mesenchymal cells in data set B. Supplementary Figure S5. Analysis of single cell RNA seq. data from data set C to show the various cellular compartments as clusters. Supplementary Figure S6. Feature plot displaying the expression of markers for various types of mesenchymal cells in data set C. Supplementary Figure S7. Feature plot displaying the expression of markers for various types of epithelial cells with the expression profile of sPLA2. Supplementary Figure S8. Feature plot displaying the expression of markers for various types of immune cells with the expression profile of sPLA2. Supplementary Figure S9. Feature plot displaying the expression of markers for various types of Endothelial cells with the expression profile of sPLA2. Supplementary Figure S10. Lung injury score/Semi-quantitative Morphological Index (SMI) in control, Bleomycin/VEH and Bleomycin/pBPB treated mice. Supplementary Figure S11. BAL fluid analysis.
