# Supplementary material for: Cellular Distribution of Secreted Phospholipase A2 in Lungs of IPF Patients and Its Inhibition in Bleomycin-Induced Pulmonary Fibrosis in Mice
Source: Cells. 2023 Mar 30;12(7):1044. doi: 10.3390/cells12071044 (PMC10092981; doi:10.3390/cells12071044)
Supplement: Supplementary file 1 [file cells-12-01044-s001.zip › Supplementary Files.pdf]

Supplementary information

Cellular Distribution of Secreted Phospholipase A2 in Lungs of IPF Patients and Its Inhibition in Bleomycin-Induced Pulmonary Fibrosis in Mice

Ashish Jaiswal [1,2,3,*], Rakhshinda Rehman [3,4], Joytri Dutta [1,2], Sabita Singh [1,2], Archita Ray [1,2], Malathy Shridhar [5], Jaswant Jaisankar [6], Manas Bhatt [6], Dikshit Khandelwal [6], Bandya Sahoo [6], Arjun Ram [3] and Ulaganathan Mabalirajan [1,2,3,*]

1 Molecular Pathobiology of Respiratory Diseases, Cell Biology and Physiology Division, Council of Scientific and Industrial Research (CSIR)—Indian Institute of Chemical Biology (IICB), Kolkata 700091, India
2 Academy of Scientific and Innovative Research (AcSIR), Sector-19, Kamla Nehru Nagar, Ghaziabad 201002, India
3 Molecular Pathobiology of Respiratory Diseases, CSIR—Institute of Genomics and Integrative Biology, Mall Road, Delhi 110007, India
4 Division of Pulmonary and Critical Care Medicine, Brigham and Women's Hospital, Boston, MA 02115, USA
5 The World Community Service Centre, Thiruvanmiyur, Chennai 600041, India
6 Kalinga Institute of Medical Sciences, KIIT University, Bhubaneswar 751024, India
* Correspondence: ashishjaiswal602@gmail.com (A.J.); mabsome@yahoo.co.in (U.M.)

Supplementary table S1

Geo accession	Gender	Tobacco	Ethnicity	Age	Diagnosis
GSM4037313	M	Yes	Caucasian	36	HEALTHY INDIVIDUAL
GSM4037309	F	No	African American	31	HEALTHY INDIVIDUAL
GSM4037323	M	Yes	Caucasian	38	HEALTHY INDIVIDUAL
GSM4037305	F	Yes	Unknown	30	HEALTHY INDIVIDUAL
GSM4037306	M	Yes	Caucasian	41	HEALTHY INDIVIDUAL
GSM4037316	M	Yes	Caucasian	54	HEALTHY INDIVIDUAL
GSM4037301	M	Yes	Caucasian	17	HEALTHY INDIVIDUAL
GSM4037302	M	Yes	Caucasian	30	HEALTHY INDIVIDUAL
GSM4037327	F	No	Caucasian	74	IPF
GSM4037319	M	Yes	Caucasian	67	IPF
GSM4037329	F	Yes	Caucasian	54	IPF
GSM4037312	F	No	Caucasian	54	IPF
GSM4037321	M	No	Caucasian	68	IPF
GSM4037322	M	No	Caucasian	63	IPF
GSM4037299	F	Yes	Caucasian	56	IPF
GSM4037320	F	No	African American	64	IPF

Supplementary Table S1. Patient’s demographic data as described by Habermann et. al. in original publication.

Supplementary table S2

Geo accession	Gender	Tobacco	Ethnicity	Age	Diagnosis
GSM3489182	F	No	African American	63	HEALTHY INDIVIDUAL
GSM3489185	M	Former	Asian	55	HEALTHY INDIVIDUAL
GSM3489187	F	No	African American	29	HEALTHY INDIVIDUAL
GSM3489191	F	Yes	White	49	HEALTHY INDIVIDUAL
GSM3489193	F	No	African American	22	HEALTHY INDIVIDUAL
GSM3489197	M	No	African American	21	HEALTHY INDIVIDUAL
GSM3489183	M	Yes	Not provided	65	IPF
GSM3489184	M	No	Not provided	60	IPF
GSM3489188	M	Yes	Not provided	68	IPF
GSM3489190	F	No	Not provided	72	IPF

Supplementary Table S2. Patient’s demographic data as described by Reyfman et. al. in original publication.

Code	Age	Gender	Smoking status	Diagnosis
Patient 1	67	F	No	IPF
Patient 2	42	F	No	IPF
Patient 3	29	F	No	IPF
Patient 4	30	F	No	IPF
Patient 5	49	F	No	IPF
Patient 6	27	F	No	IPF
Patient 7	49	F	No	IPF
Patient 8	42	F	No	IPF
Patient 9	52	F	No	IPF
Patient 10	54	F	No	IPF
Control 1	31	F	No	Healthy individual
Control 2	28	F	No	Healthy individual
Control 3	47	F	No	Healthy individual
Control 4	40	F	No	Healthy individual
Control 5	47	F	No	Healthy individual
Control 6	50	F	No	Healthy individual
Control 7	41	F	No	Healthy individual
Control 8	38	F	No	Healthy individual
Control 9	44	F	No	Healthy individual
Control 10	48	F	No	Healthy individual
Control 11	39	F	No	Healthy individual
Control 12	52	F	No	Healthy individual
Control 13	42	F	No	Healthy individual
Control 14	49	F	No	Healthy individual
Control 15	32	F	No	Healthy individual
Control 16	39	F	No	Healthy individual
Control 17	40	F	No	Healthy individual
Control 18	41	F	No	Healthy individual
Control 19	39	F	No	Healthy individual
Control 20	51	F	No	Healthy individual

Supplementary Table S3. Demographic profile of patients and healthy individuals utilized for measuring sPLA2-IIA in human sera of both IPF patients and controls.

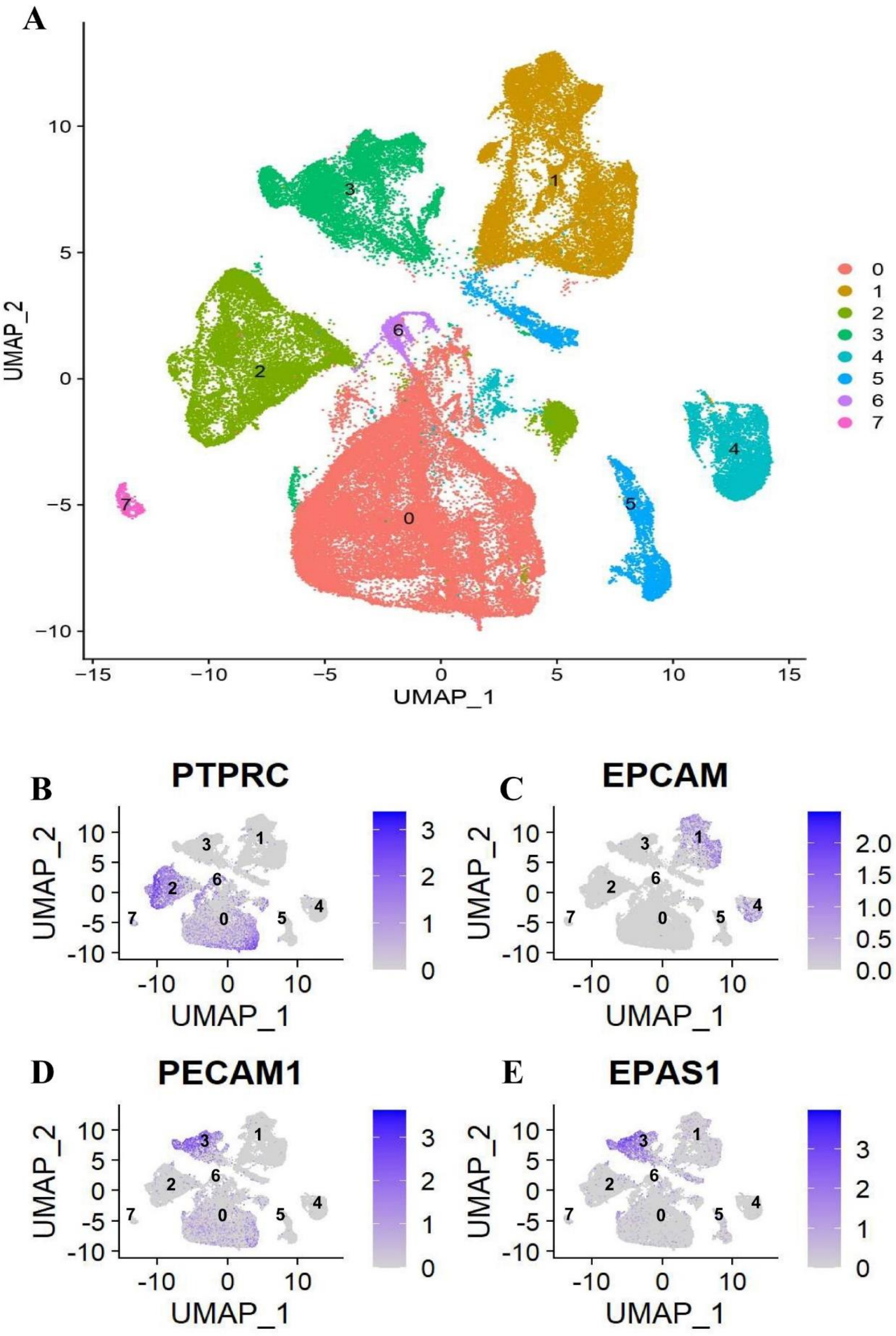

Supplementary Figure S1. Analysis of single cell RNA seq. data **from data set A to show the various cellular compartments as clusters.** (A) Uniform Manifold Approximation of projection (UMAP) after analysing Single cell RNA seq. data from 8 Control (healthy

individuals) and 8 IPF patients, using Seurat as described by Habermann et al. Feature plots displaying the expression canonical markers of Immune cells (PTPRC+ cells, 0, 2, 6 and 7) (B), Epithelial cells (EPCAM+ cells, cluster 1 and 4) (C) and, Endothelial cells (PECAM1+ and EPAS1+ cells, cluster3) (D & E). Mesenchymal cells were EPCAM, PTPRC, and PECAM1 negative cells, cluster 5).

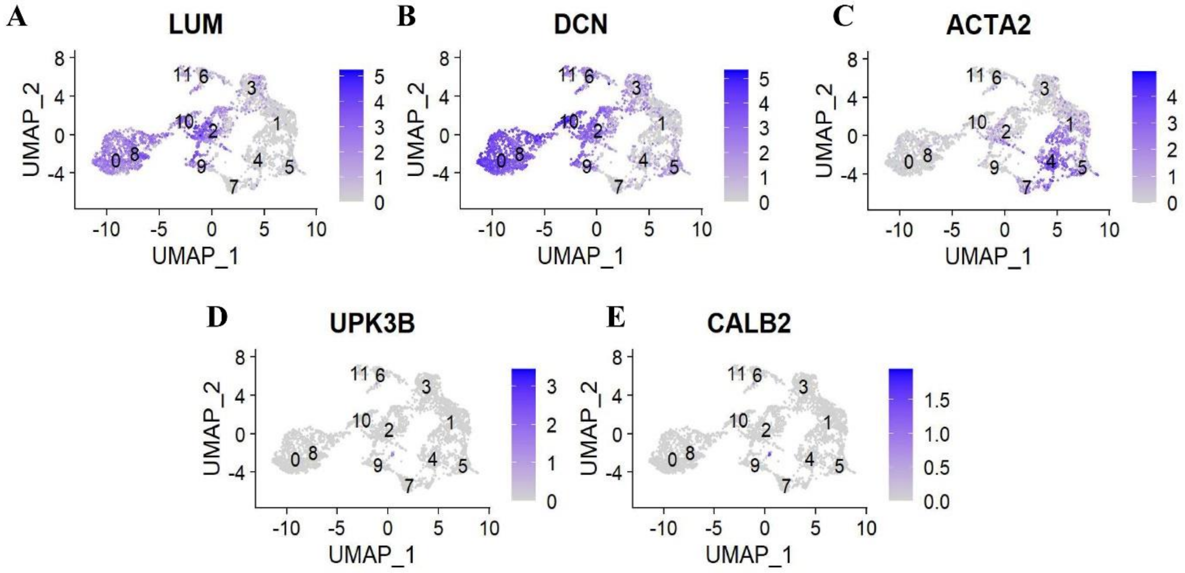

Supplementary Figure S2. Feature plot displaying the expression of markers for various types of Mesenchymal cells in data set A: (A-C) DCN high fibroblast-LUM+ and DCN high cells (cluster 9 and 10) and DCN low fibroblast-LUM+ and DCN low cells (cluster 3, 11, 6), Myofibroblast- both LUM+ and ACTA2+ cells (cluster 2), PLA2G2A IPF fibroblasts-LUM and PLA2G2A high cells (cluster 0 and 8), Smooth muscle cells-ACTA2+ cells (1, 4, 5 and 7) and (D-E) mesothelial cells-UPK3B+ and CALB2+ cells.

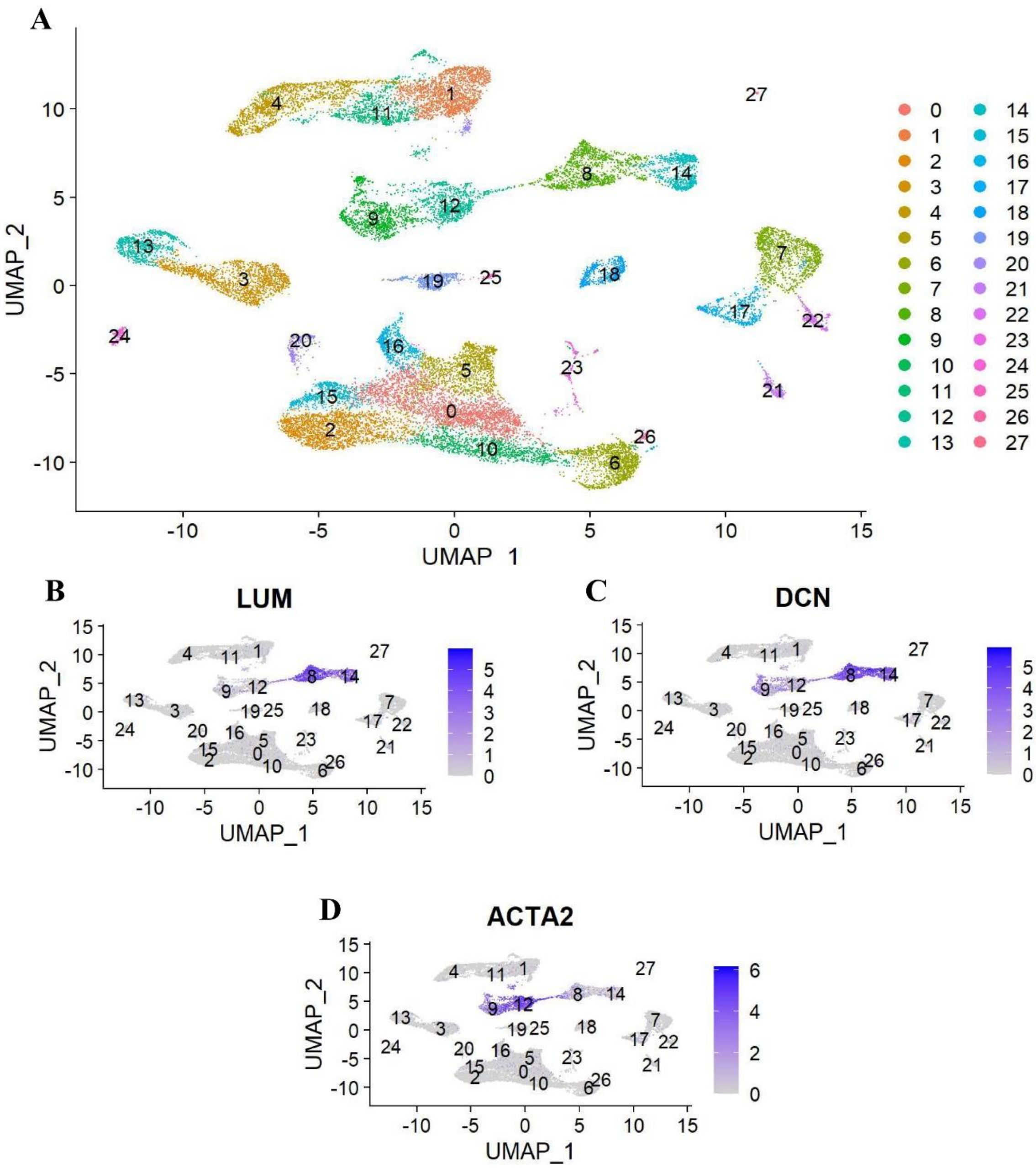

Supplementary Figure S3. Analysis of single cell RNA seq. data from data set B to show the various cellular compartments as clusters. (A) Uniform Manifold Approximation of projection (UMAP) after analysing Single cell RNA seq. data from 3 Control (healthy individuals) and 3 IPF patients, using Seurat as described by Tsukui et al. (B-D) Feature plot showing the expression of markers of mesenchymal cells (Fibroblasts and smooth muscle cells).

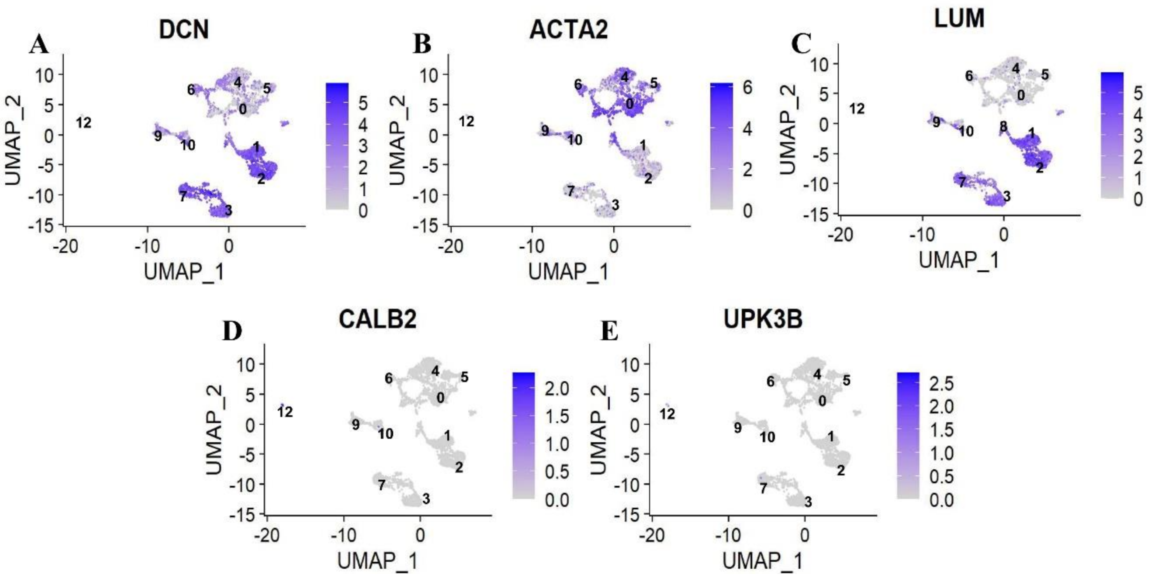

Supplementary Figure S4. Feature plot displaying the expression of markers for various types of Mesenchymal cells in data set B: (A-E) Various mesenchymal cells are identified by the expression of canonical markers, DCN and LUM+ cells- fibroblast Myofibroblast-both LUM+ and ACTA2+ cells, PLA2G2A IPF fibroblasts-LUM and PLA2G2A high cells, Smooth muscle cells-ACTA2+ cells and mesothelial cells UPK3B and CALB2+ cells.

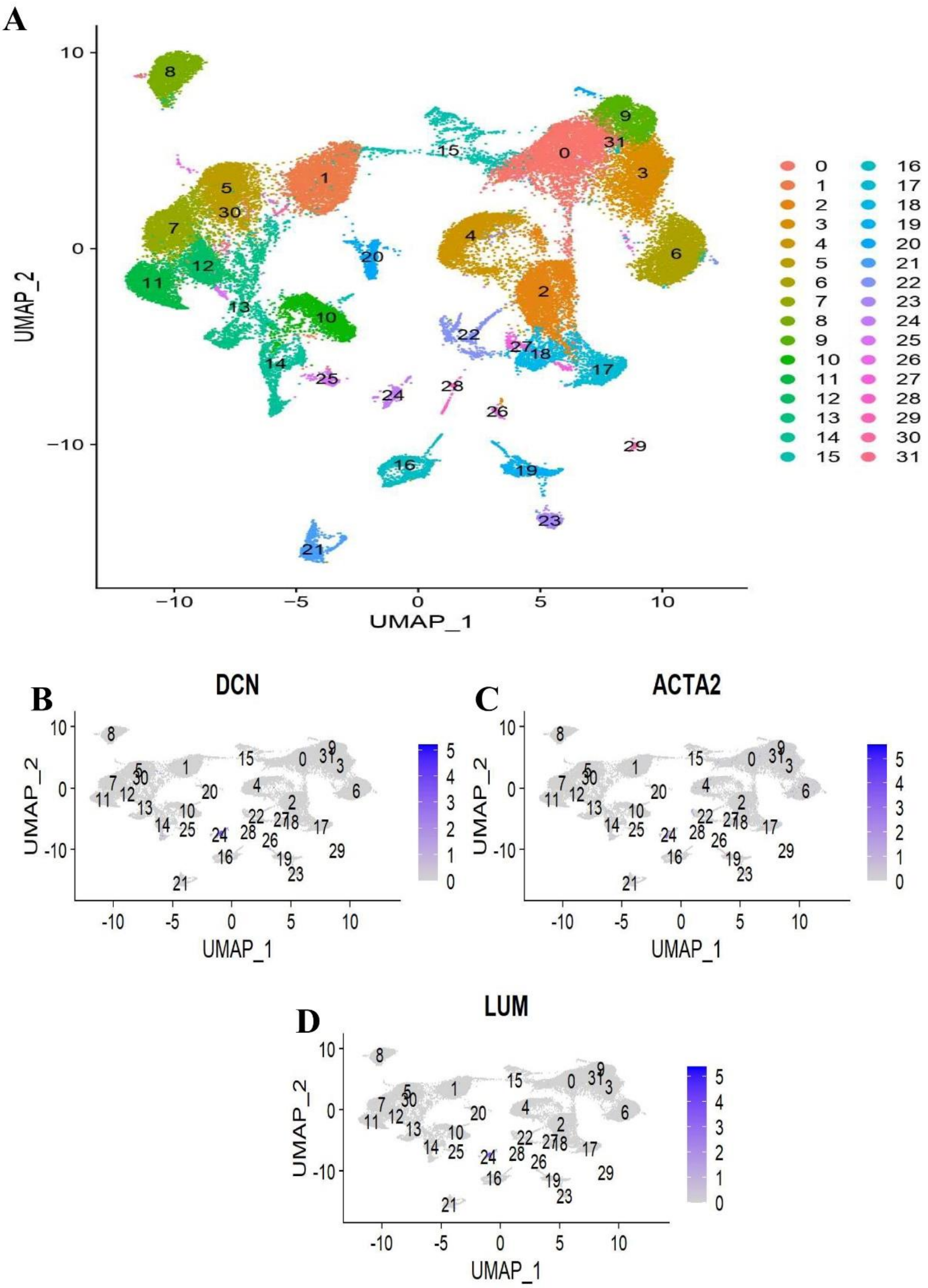

Supplementary Figure S5. Analysis of single cell RNA seq. data **from data set C to show the various cellular compartments as clusters.** (A) Uniform Manifold Approximation of projection (UMAP) after analysing Single cell RNA seq. data from 8 Control (healthy individuals) and 4 IPF patients, using Seurat as described by Reyfman et al. (B-D) Feature plot showing the expression of markers of mesenchymal cells (Fibroblasts and smooth muscle cells).

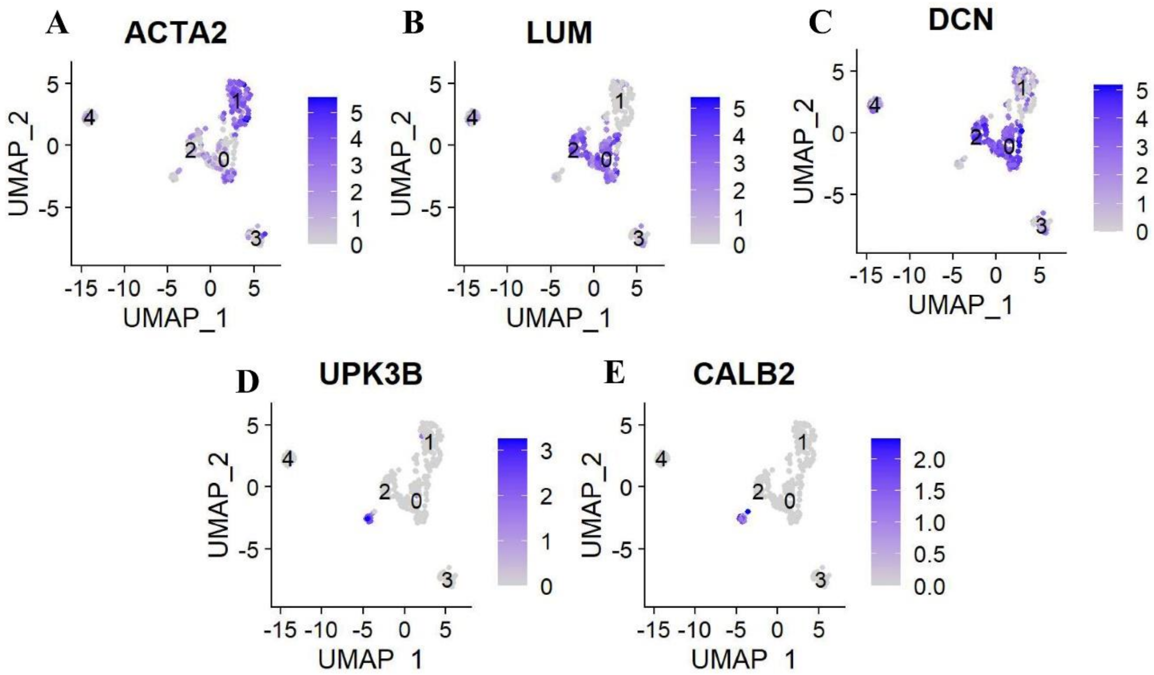

Supplementary Figure S6. Feature plot displaying the expression of markers for various types of Mesenchymal cells in data set C:

Various mesenchymal cells are identified by the expression of canonical markers, DCN and LUM+ cells- fibroblast Myofibroblast- both LUM+ and ACTA2+ cells, PLA2G2A IPF fibroblasts-LUM and PLA2G2A high cells, Smooth muscle cells-ACTA2+ cells and mesothelial cells UPK3B and CALB2+ cells.

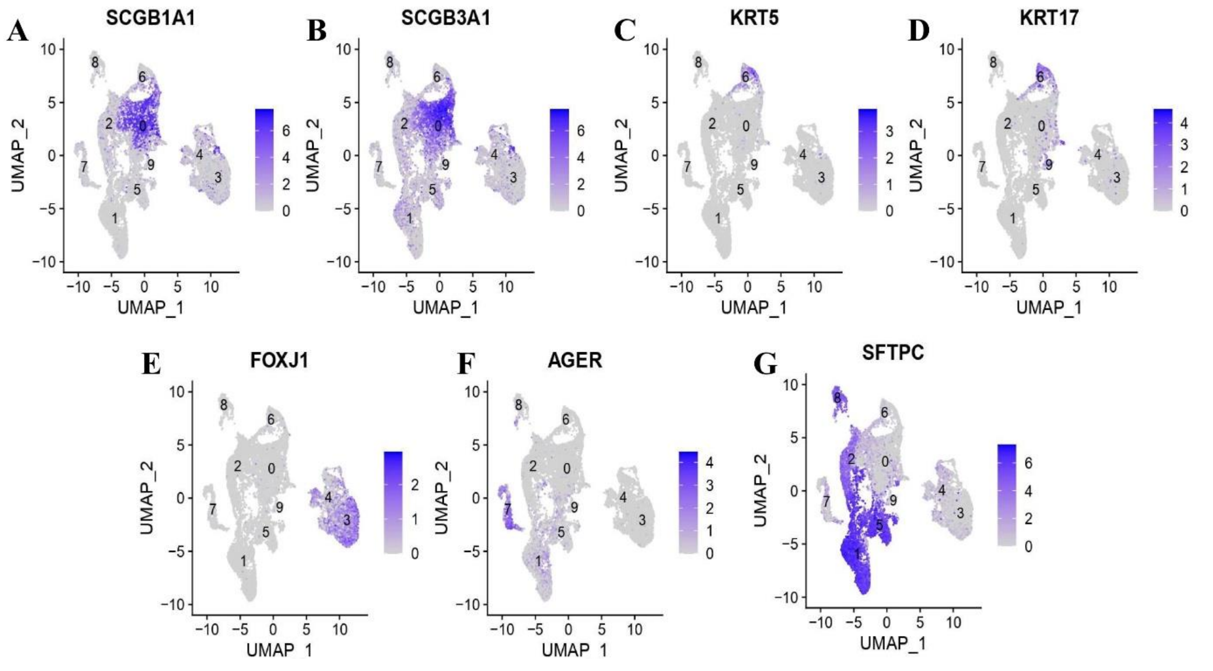

Supplementary figure S7. Feature plot displaying the expression of markers for various types of Epithelial cells with the expression profile of sPLA2: (A-G) Club cells-SCGB1A1 and SCGB3A1+ cells (cluster 0), Basal cells-KRT5 and KRT17+ cells (cluster 6), KRT5-/KRT17+ cells-KRT5 negative and KRT17+ cells (cluster 9), Ciliated cells-FOXJ1+ cells (cluster 3 and 4); AT1 cells (alveolar type1 cells)-AGER+ cells (cluster 7) and AT2 (alveolar type2 cells)-SFTPC+ cells (cluster 1, 2, 5, 8).

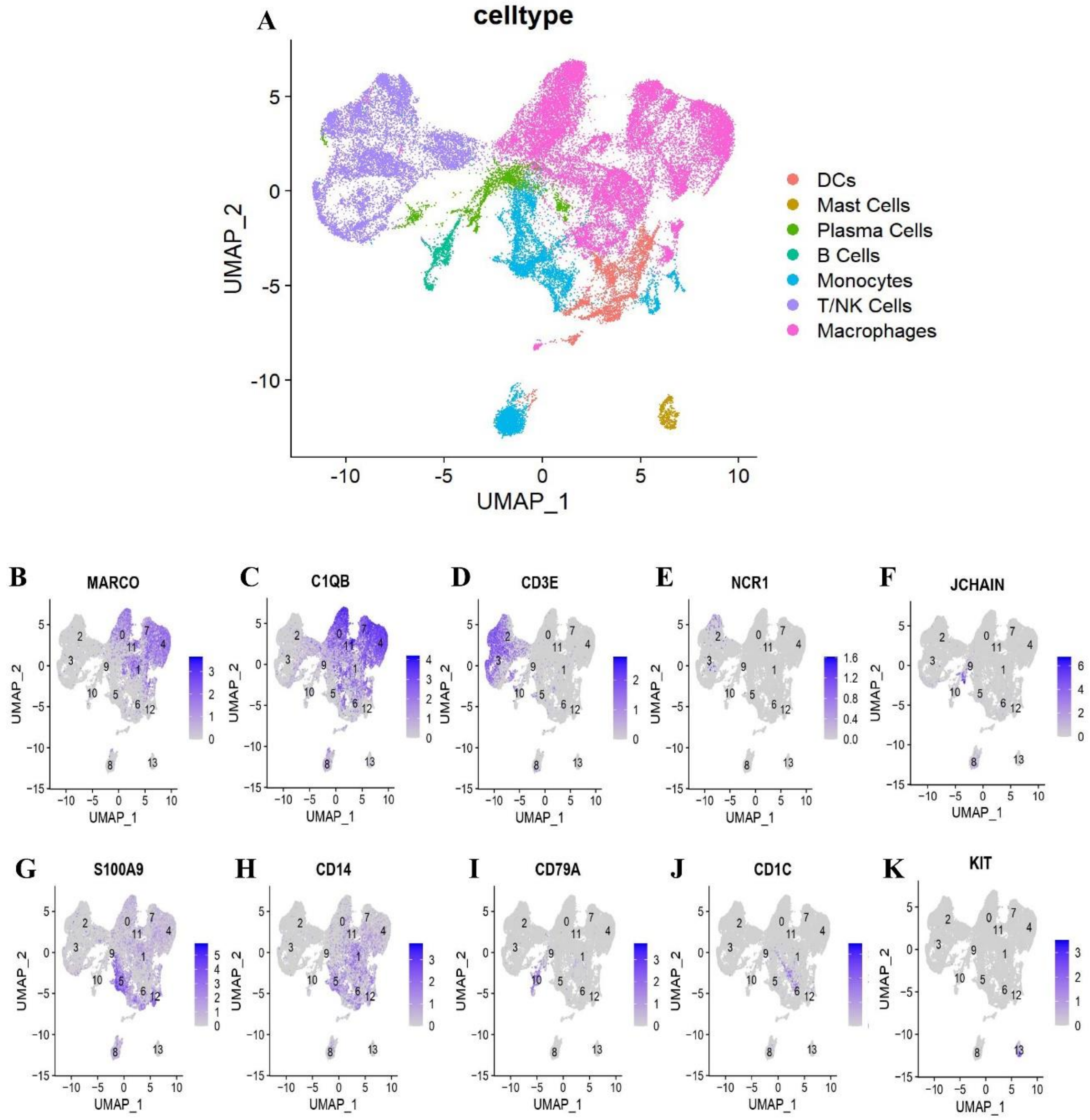

Supplementary figure S8. Feature plot displaying the expression of markers for various types of Immune cells with the expression profile of sPLA2: (A) UMAP displaying various clusters of immune cells after annotation, (B-J) Macrophages-MARCO and C1QB+ cells (cluster 0, 1, 4, 7, and 11), T/NK cells-CD3E/NCR1+ cells (cluster 2, 3), Plasma cells-JCHAIN+ cells (cluster 9), Monocytes-CD14+ and S100A9+ cells (cluster 5, 12 and 8), , B

cells-CD79A+ cells (cluster 10), DCs-CD1C+ cells (cluster 6), Mast cells-KIT+ cells (cluster 13).

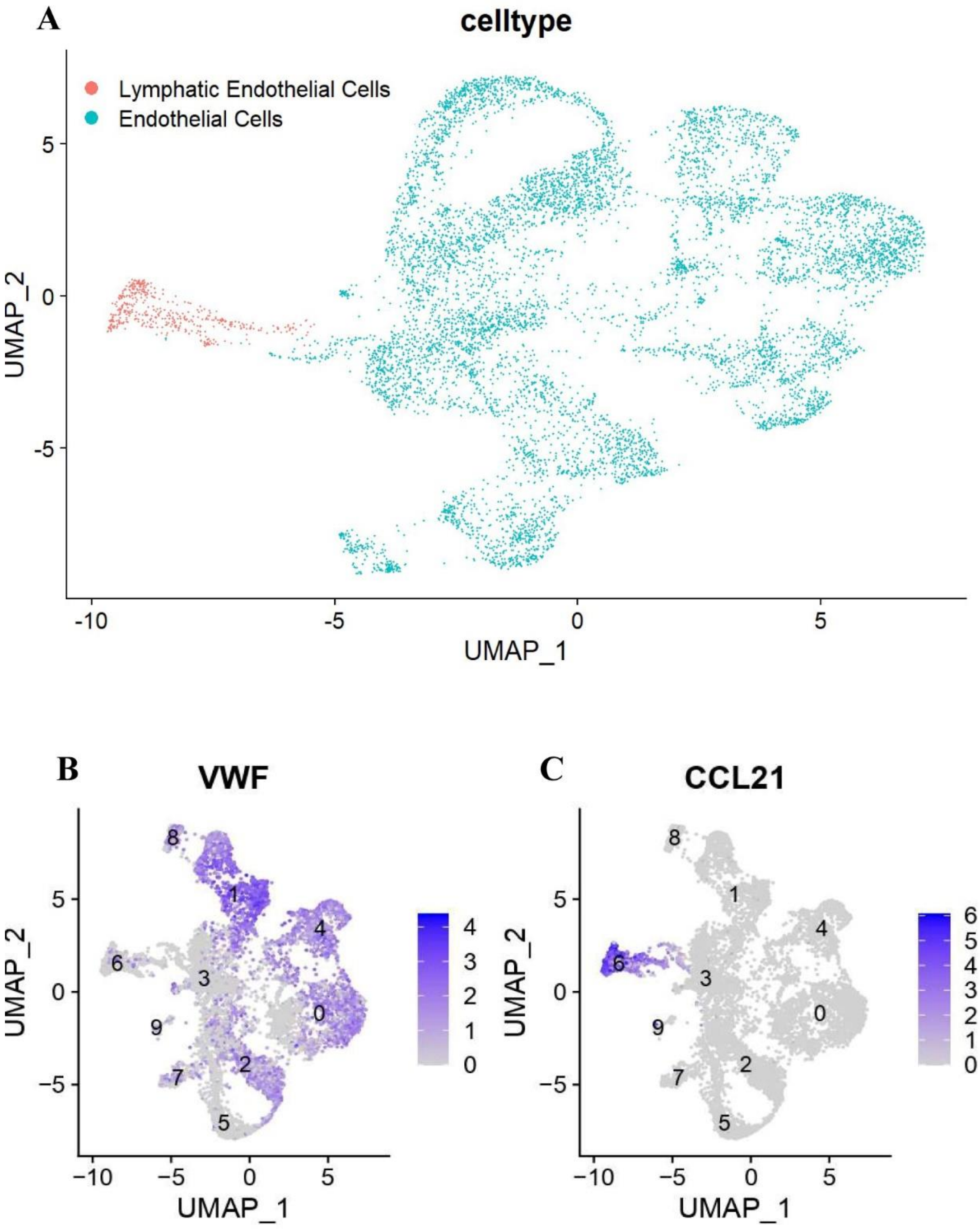

Supplementary figure S9. Feature plot displaying the expression of markers for various types of Endothelial cells with the expression profile of sPLA2: (A) UMAP displaying various clusters of endothelial cells after annotation, (B) Endothelial cells-VWF+ cells (cluster 0, 1, 2, 3, 4, 5, 7, 8 and 9), Lymphatic endothelial cells-CCL21+ cells (cluster 6).

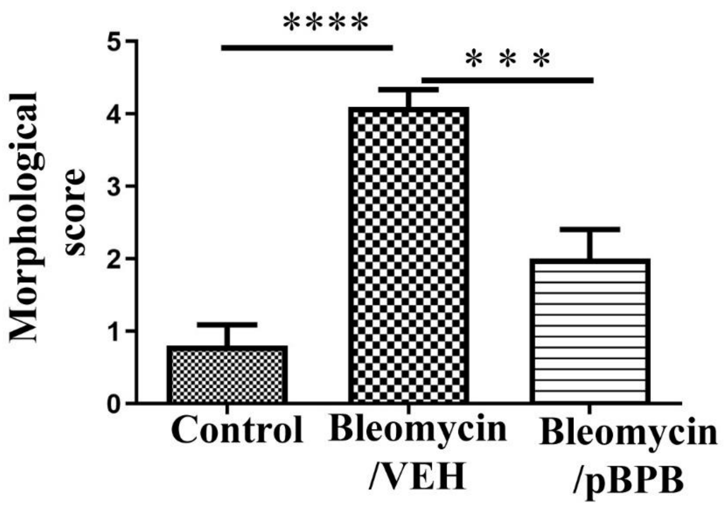

Supplementary figure S10. Lung injury score/Semi-quantitative Morphological Index (SMI) in Control, Bleomycin/VEH and Bleomycin/pBPB treated mice. Detailed method for measuring SMI is discussed in methods section. p=0.0001 for Control verses Bleomycin/VEH group and p= 0.0009 for Bleomycin/VEH verses Bleomycin/pBPB group.

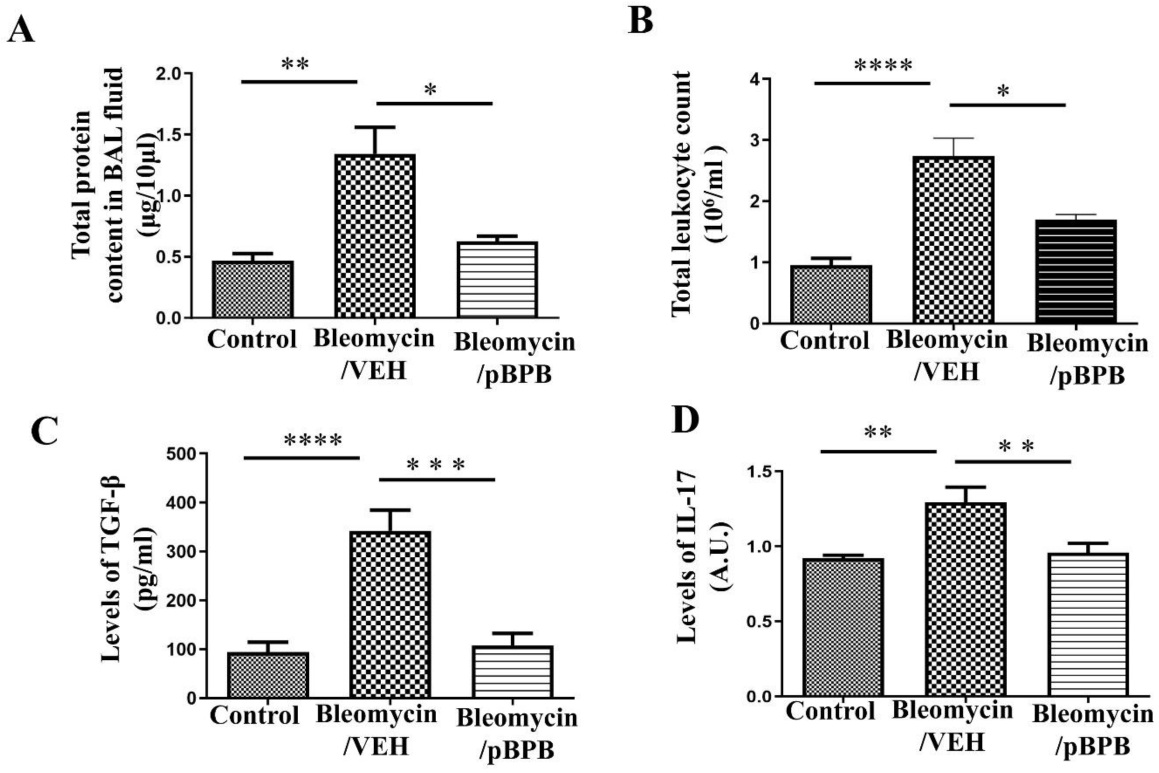

Supplementary figure S11. BAL fluid analysis: (A) Total protein content in BAL fluid of Control, Bleomycin/VEH and Bleomycin/pBPB treated mice. (B) Total leukocytes count in the BAL fluid of Control, Bleomycin/VEH and Bleomycin/pBPB treated mice (C) & (D) TGF-β and IL-17 ELISA in the BAL fluid of Control, Bleomycin/VEH and Bleomycin/pBPB treated mice.
